# Structural Evolution of RAFT-Modified Unsaturated Polyester Copolymers: Effects of CPDT Concentration, Acidic Comonomer Structure, and Polyester Matrix Architecture

**DOI:** 10.3390/molecules31172958

**Published:** 2026-08-24

**Authors:** Meruyert S. Zhunissova, Akmaral Zh. Sarsenbekova, Altynaray T. Takibayeva, Tolkyn O. Khamitova, Aigerim Zhaxybayeva, Saltanat Kaliyeva, Balken Kuderina, Gulnaz N. Musina, Akkenzhe Bussurmanova

**Affiliations:** 1 Department of Physical and Analytical Chemistry, Faculty of Chemistry, Karaganda Buketov National Research University, Karaganda 100024, Kazakhstan; meruert_zhunusova@mail.ru; 2 Department of Chemical Technology and Ecology, Faculty of Metallurgy and Mechanical Engineering, Karaganda Industrial University, Temirtau 101400, Kazakhstan; 3 Institute of Agriculture and Forestry, Saken Seifullin Kazakh Agrotechnical University, Astana 010000, Kazakhstan; 4 Department of Chemistry, Institute of Natural Sciences, L.N. Gumilyov Eurasian National University, Astana 010008, Kazakhstan; 5 School of Natural Sciences, Astana International University, Astana 010000, Kazakhstan; 6 Department of Morphology, Physiology and General Pathology, Higher School of Medicine, Ualikhanov University, Kokshetau 020000, Kazakhstan; 7 Department of Chemistry and Chemical Technology, Faculty of Metallurgy and Mechanical Engineering, Abylkas Saginov Karaganda Technical University, Karaganda 100027, Kazakhstan; 8 Department of Natural Sciences, Faculty of Pedagogy, Yessenov University, Aktau 130000, Kazakhstan

**Keywords:** RAFT polymerization, unsaturated polyester resins, CPDT, ^1^H NMR spectroscopy, ^1^H–^1^H COSY, UV–Vis spectroscopy, gel permeation chromatography, molecular-weight distribution, acrylic acid, methacrylic acid

## Abstract

Unsaturated polyester resins (UPRs) represent challenging systems for reversible-deactivation radical polymerization (RDRP) because chain propagation, branching, and localized gelation may occur concurrently. This study systematically investigates the influence of the concentration of the RAFT agent 2-cyano-2-propyl dodecyl trithiocarbonate (CPDT), the chemical structure of the polyester prepolymer, and the nature of the acidic comonomer on the structural evolution of RAFT-modified unsaturated polyester copolymers. Three copolymer series synthesized at different CPDT concentrations were investigated: p-EGM:AA:[CPDT], p-EGM:MAA:[CPDT], and p-PGM:MAA:[CPDT]. Structural changes were characterized using H NMR, H–H COSY, UV–Vis spectroscopy, and gel permeation chromatography (GPC). Semi-quantitative analysis of normalized H NMR integral intensities was performed using Relative Vinyl Intensity (*RVI*), CPDT-associated methyl intensity (MI*), and normalized aliphatic intensity (AI*) to compare changes in selected proton environments among the investigated copolymer series. Increasing CPDT concentration was accompanied by a decrease in the normalized residual maleate vinyl signal, although the magnitude of this change depended strongly on copolymer composition. The most pronounced decrease in *RVI* was observed for the p-EGM:AA:[CPDT] series, from 0.6291 to 0.0528, whereas substantially smaller changes were observed for the p-EGM:MAA:[CPDT] series. The MI* and AI* profiles exhibited composition-dependent variations, reflecting changes in the relative contributions of CPDT-associated methyl and overlapping aliphatic proton environments, respectively. Because the aliphatic region used for AI* contains overlapping polymer- and CPDT-derived contributions, AI* is not interpreted as a quantitative measure of polymer-backbone branching. Overall, the combined NMR and GPC/SEC results reveal composition-dependent structural changes accompanying RAFT copolymerization and demonstrate that both the polyester matrix and the acidic comonomer influence the response of these heterogeneous unsaturated polyester systems to variations in CPDT concentration.

## 1. Introduction

Unsaturated polyester resins (UPRs) represent one of the most commercially and technologically important classes of thermosetting polymers because of their favorable combination of mechanical strength, chemical resistance, processability, and cost-effectiveness [1,2,3]. Reactive double bonds incorporated into the polyester backbone enable radical copolymerization with vinyl monomers, producing materials with widely tunable physicochemical and performance properties [1,2,3]. Consequently, UPRs are extensively used in structural composites, protective coatings, adhesives, and other engineering applications [1,2,3].

Despite their widespread industrial use, conventional free-radical copolymerization of UPRs provides limited control over the resulting macromolecular architecture and network topology. Rapid chain propagation, accompanied by chain-transfer and irreversible termination reactions, typically produces materials with broad molecular-weight distributions, pronounced structural heterogeneity, and poorly controlled macromolecular organization [4,5,6]. Achieving improved structural and topological control during the formation of UPR-based materials therefore remains an important challenge in contemporary polymer chemistry [4,5,6].

Among reversible-deactivation radical polymerization (RDRP) techniques, reversible addition–fragmentation chain-transfer (RAFT) polymerization is one of the most versatile strategies for controlling macromolecular architecture [7,8,9,10]. RAFT control is mediated by a rapid dynamic equilibrium involving addition–fragmentation reactions between propagating radicals and thiocarbonylthio-containing dormant chains. This mechanism promotes more uniform chain growth and can provide control over molecular weight, molecular-weight distribution, and chain-end functionality under suitable polymerization conditions [7,8,9,10]. Accordingly, RAFT polymerization has been widely applied to the synthesis of functional vinyl polymers for diverse advanced applications [7,8,9,10,11].

However, whereas most RAFT studies concern linear vinyl homopolymers and copolymers, the application of this approach to unsaturated polyester matrices remains comparatively underexplored [8,9,10,11,12,13]. Unlike conventional monofunctional vinyl monomers, UPRs contain reactive double bonds incorporated directly into the polyester main chain. Their radical copolymerization can therefore generate a complex combination of chain growth, branching, and progressive cross-linking, resulting in structurally heterogeneous materials containing soluble branched species together with partially cross-linked or gelled domains [14,15]. This structural heterogeneity makes it difficult to regulate molecular-weight development and the evolution of macromolecular architecture using conventional free-radical polymerization alone. In this context, RAFT is particularly attractive not simply as an alternative polymerization technique, but as a means of regulating radical chain growth before extensive network formation occurs. By controlling the balance between propagation and reversible chain transfer, RAFT has the potential to influence the development of soluble branched chains, residual unsaturation, and the subsequent evolution of the cross-linked structure. Such control is important because the molecular architecture established during polymerization can influence the thermal behavior and, potentially, the bulk performance of UPR-based materials. Therefore, understanding how RAFT-agent concentration affects structural evolution in these inherently heterogeneous systems is an important step toward establishing structure–processing–property relationships in RAFT-modified thermosetting polyester materials.

Despite growing interest in RAFT-modified UPRs, the extent to which the chemical structure of the polyester prepolymer and the nature of the acidic comonomer determine the resulting copolymer architecture remains insufficiently understood. Previous studies have mainly focused on polymerization kinetics, synthetic procedures, and macroscopic material properties [16,17], whereas the relationships among polyester structure, RAFT-agent concentration, residual unsaturation, and molecular-weight characteristics have received considerably less attention [14,15,18].

In our previous study, we demonstrated that variation in the concentration of the RAFT agent 2-cyano-2-propyl dodecyl trithiocarbonate (CPDT) significantly affected the thermal behavior and decomposition kinetics of RAFT-modified copolymers based on poly(propylene glycol maleate) and acrylic acid [19]. Although the results indicated that differences in macromolecular architecture strongly influenced the thermal-degradation pathways, the molecular origins of these effects and the structural processes occurring during copolymer formation were not fully elucidated [19].

The characterization of such inherently heterogeneous, branched, and partially cross-linked systems requires a comprehensive analytical approach. Complementary spectroscopic and chromatographic techniques are therefore needed to evaluate both the chemical structure of the copolymers and the molecular-weight characteristics of their soluble fractions [20,21]. Accordingly, molecular-weight data obtained by GPC should be interpreted specifically as characteristics of the soluble copolymer fraction rather than of the entire partially cross-linked material.

The present work therefore investigates how the concentration of the CPDT RAFT agent, the chemical structure of the polyester matrix, and the nature of the acidic comonomer govern the structural evolution of RAFT-modified polyester copolymers synthesized from previously developed UPRs [22,23]. A combination of complementary analytical techniques, including H NMR spectroscopy, UV–Vis spectroscopy, and gel permeation chromatography (GPC), was employed to establish relationships among residual unsaturation, molecular-weight characteristics, and RAFT-mediated structural organization. Particular attention was given to the soluble branched copolymer fraction accessible to GPC analysis, while recognizing that the investigated materials also contain partially cross-linked domains. By identifying how RAFT concentration and monomer structure influence molecular-level organization prior to and during network development, this study provides a structural basis for understanding the previously observed differences in thermal behavior and represents a step toward establishing structure–property relationships in RAFT-modified UPR materials.

## 2. Results and Discussion

### 2.1. Soluble and Insoluble Fraction Analysis

Gravimetric analysis of the isolated soluble and insoluble fractions revealed a pronounced dependence of the product distribution on CPDT concentration. The complete gravimetric mass-balance data, including the initial mass, dry masses of the soluble and insoluble fractions, total recovered mass, overall recovery, and the corresponding soluble and insoluble mass fractions, are provided in Table S1. At the lowest CPDT concentrations (10.01–10.02 mM), the recovered products were predominantly insoluble, with insoluble fractions of 95.98–96.15% and soluble fractions of only 3.85–4.02%. Increasing the CPDT concentration progressively increased the proportion of soluble material. At approximately 30 mM CPDT, the soluble fraction increased to 14.57–14.67%, whereas at approximately 50 mM the soluble and insoluble fractions became nearly comparable, with the soluble fraction reaching 48.51–48.90%. At the highest CPDT concentrations (80.03–80.05 mM), the soluble fraction constituted 93.82–94.78% of the recovered product.

This trend was observed consistently for the p-EGM:AA:[CPDT], p-EGM:MAA:[CPDT], and p-PGM:MAA:[CPDT] series, indicating that CPDT concentration strongly affects the partitioning of the recovered reaction product between soluble and insoluble fractions. The predominance of the insoluble fraction at low CPDT concentrations is consistent with, rather than direct quantitative evidence of, more extensive network formation under these conditions. Conversely, the marked increase in the soluble fraction at higher CPDT concentrations is consistent with reduced formation of insoluble network structures under conditions of greater chain-transfer-agent availability [14,15].

Importantly, these gravimetric results also define the scope of the spectroscopic and chromatographic characterization. The NMR, COSY, UV–Vis, and GPC/SEC measurements reported in this study were performed on the isolated soluble fractions. Consequently, particularly at low CPDT concentrations, where the soluble fraction represents only approximately 4% of the recovered material, these measurements should not be interpreted as quantitatively representative of the entire heterogeneous reaction product. At higher CPDT concentrations, the soluble fraction becomes the predominant recovered fraction and is therefore substantially more representative of the recovered product.

It should be emphasized that component-specific conversions of the polyester maleate units and AA or MAA were not independently determined in the present study. The gravimetric soluble/insoluble fraction analysis reported in Table S1 describes the partitioning of the recovered reaction product and does not provide separate monomer conversions. Likewise, the normalized residual maleate vinyl intensities obtained by ^1^H NMR were determined for the isolated soluble fractions and are therefore used only as semi-quantitative descriptors of residual unsaturation rather than as absolute maleate conversion values for the entire heterogeneous reaction system. Accordingly, separate conversions of the polyester maleate units and AA/MAA cannot be reliably derived from the available measurements. This limitation should be considered when interpreting the CPDT-concentration-dependent structural changes discussed below.

### 2.2. Structural Characterization

#### 2.2.1. Characteristic NMR Signals

**Figure 1:**
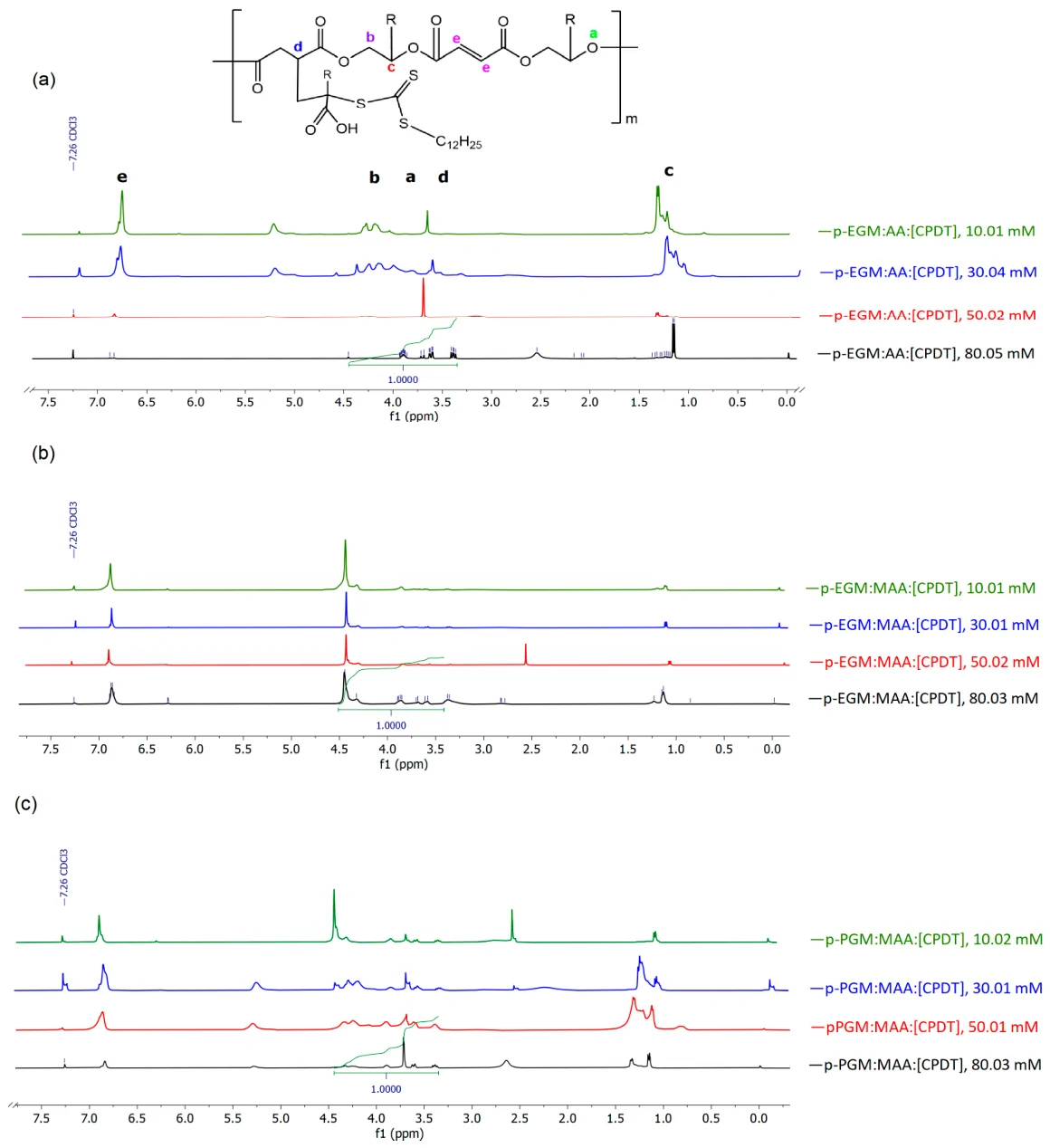
Representative ^1^H NMR spectra of RAFT copolymers synthesized at different CPDT concentrations: (**a**) p-EGM:AA:[CPDT] at 10.01, 30.04, 50.02, and 80.05 mM; (**b**) p-EGM:MAA:[CPDT] at 10.01, 30.01, 50.02, and 80.03 mM; and (**c**) p-PGM:MAA:[CPDT] at 10.02, 30.01, 50.01, and 80.03 mM. In each panel, the green, blue, red, and gray spectra correspond to increasing CPDT concentrations, respectively. The lowercase letters a–e in the representative polymer structure denote chemically distinct proton environments and correspond to the lettered signal assignments in the ^1^H NMR spectra. The font colors of these letters are used solely for visual distinction and do not represent an additional experimental variable. Signal a represents predominantly –O–CH_2_– and other overlapping oxygenated proton environments of the polyester backbone (*δ* = 3.35–4.44 ppm); signals b–d represent overlapping aliphatic proton environments associated with polyester- and comonomer-derived structural fragments; and signal e is assigned to residual maleate vinyl protons (*δ* = 6.75–6.95 ppm). The residual CHCl_3_ solvent signal from CDCl_3_ is observed at *δ* ≈ 7.26 ppm. Owing to extensive signal overlap in the aliphatic and polyester-backbone regions, these assignments should be regarded as approximate spectral-region assignments rather than uniquely resolved individual proton resonances.

To evaluate the influence of the CPDT RAFT agent concentration on the structural characteristics of the synthesized copolymers, the ^1^H NMR spectra of the three investigated copolymer series were systematically compared. Representative spectra are presented in Figure 1, whereas the complete ^1^H and ^13^C NMR spectra of all synthesized samples are provided in the Supplementary Materials (Figures S1–S3).

Across the investigated range of CPDT concentrations, the principal characteristic resonances remained identifiable within the same general spectral regions (Figure 1), indicating that the major proton environments characteristic of the copolymer systems were retained. However, the spectra are not identical. Variations in the apparent positions, line shapes, relative intensities, and splitting patterns of several resonances are observed, particularly in the spectrum obtained at the highest CPDT concentration in Figure 1a. These differences may reflect changes in the local proton environments and/or in the extent of resonance overlap within the isolated soluble copolymer fraction. Because several resonances are strongly overlapped, particularly in the polyester-backbone and aliphatic regions, these variations cannot be unambiguously attributed to a single structural transformation and are therefore interpreted qualitatively.

The weak resonances in the *δ* = 6.75–6.95 ppm region (signal e, Figure 1) were assigned to residual maleate vinyl protons. The *δ* = 3.35–4.44 ppm region contains predominantly O–CH_2_ and other overlapping oxygenated proton environments of the polyester backbone, represented by signal a in Figure 1. Signals b–d represent overlapping aliphatic proton environments associated with polyester- and comonomer-derived structural fragments. Owing to extensive signal overlap, these assignments should be regarded as approximate spectral-region assignments rather than as uniquely resolved individual proton resonances. The residual CHCl_3_ solvent signal from CDCl_3_ was observed at *δ* ≈ 7.26 ppm and is explicitly indicated in Figure 1.

For the semi-quantitative comparison, the integral intensity of the polyester-backbone region at *δ* = 3.35–4.44 ppm was used as the normalization reference and set to unity (*I*_norm_ = 1.00). This normalization enabled comparison of the relative integral intensities of selected spectral regions among the investigated samples while minimizing the influence of differences in absolute spectral intensity. The most pronounced changes were observed in the residual maleate vinyl region at *δ* = 6.75–6.95 ppm. Accordingly, changes in the normalized intensity of this region were used as a comparative spectral descriptor of changes in residual unsaturation with increasing CPDT concentration.

#### 2.2.2. ^1^H–^1^H COSY Spectroscopy

**Figure 2:**
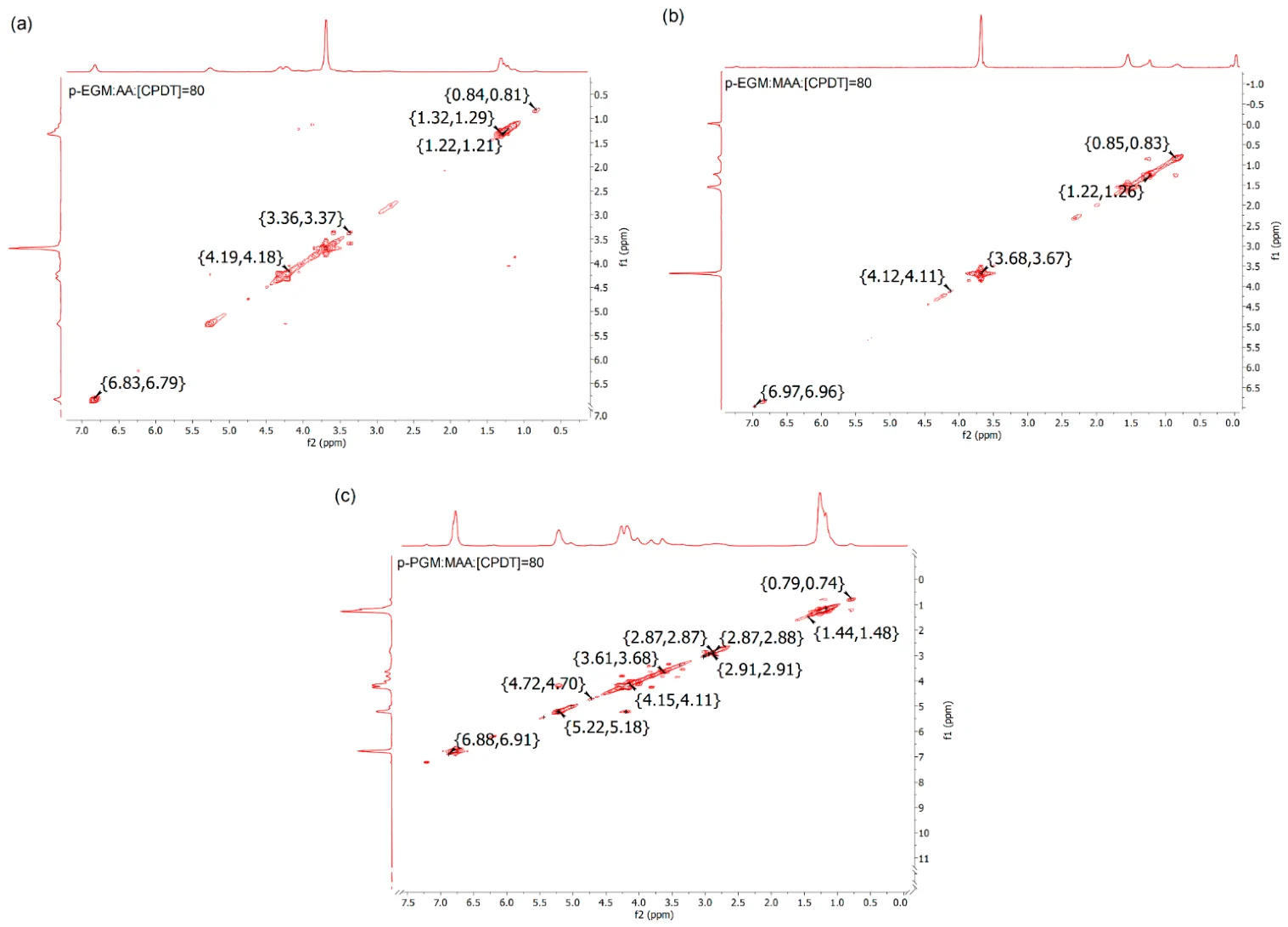
Representative ^1^H–^1^H COSY spectra of RAFT copolymers synthesized at [CPDT] = 80 mM: (**a**) p-EGM:AA, (**b**) p-EGM:MAA, and (**c**) p-PGM:MAA. The spectra reveal the principal through-bond correlations between neighboring proton environments.

To confirm the signal assignments established by one-dimensional (^1^H) NMR spectroscopy, two-dimensional ^1^H–^1^H COSY spectra were recorded for representative samples from each of the three investigated RAFT copolymer series synthesized at a CPDT concentration of 80.03 mM (Figure 2).

A comparison of the COSY spectra of p-EGM:AA:[CPDT] (Figure 2a) and p-EGM:MAA:[CPDT] (Figure 2b) demonstrates the influence of the acidic comonomer on the macromolecular structure while maintaining the same EGM polyester matrix. In both spectra, the characteristic cross-peaks of the polyester backbone in the *δ* = 3.30–4.20 ppm region are preserved, confirming that the polyester framework remains structurally intact. In contrast, the cross-peaks assigned to the residual maleate double bonds shift from *δ* = 6.79–6.83 ppm for p-EGM:AA:[CPDT] (Figure 2a) to *δ* = 6.96–6.97 ppm for p-EGM:MAA:[CPDT] (Figure 2b). This downfield shift is likely associated with the additional α-methyl group of the methacrylic acid units, which modifies the electronic and steric environment of the neighboring structural fragments. Differences observed in the aliphatic region (*δ* = 0.80–1.30 ppm) further reflect the distinct chemical environments generated by the two acidic comonomers.

The influence of the polyester matrix becomes more evident when comparing the COSY spectra of p-EGM:MAA:[CPDT] (Figure 2b) and p-PGM:MAA:[CPDT] (Figure 2c). In the PGM-based copolymer, additional correlation cross-peaks appear at *δ* = 2.87–2.91 ppm and *δ* = 1.44–1.48 ppm, showing correlations with signals in the *δ* = 4.15–4.72 ppm region. These cross-peaks are assigned to the propylene glycol units of the polyester chain and reflect the distinct local chemical environment resulting from replacing the ethylene glycol-based polyester matrix with the corresponding propylene glycol analogue. Nevertheless, the principal correlation pathways of the polyester backbone (*δ* = 3.60–4.20 ppm) together with the weak correlations of the residual maleate vinyl protons (*δ* = 6.80–7.00 ppm) remain preserved in all three investigated copolymer series (Figure 2).

#### 2.2.3. Relative Vinyl Intensity

**Figure 3:**
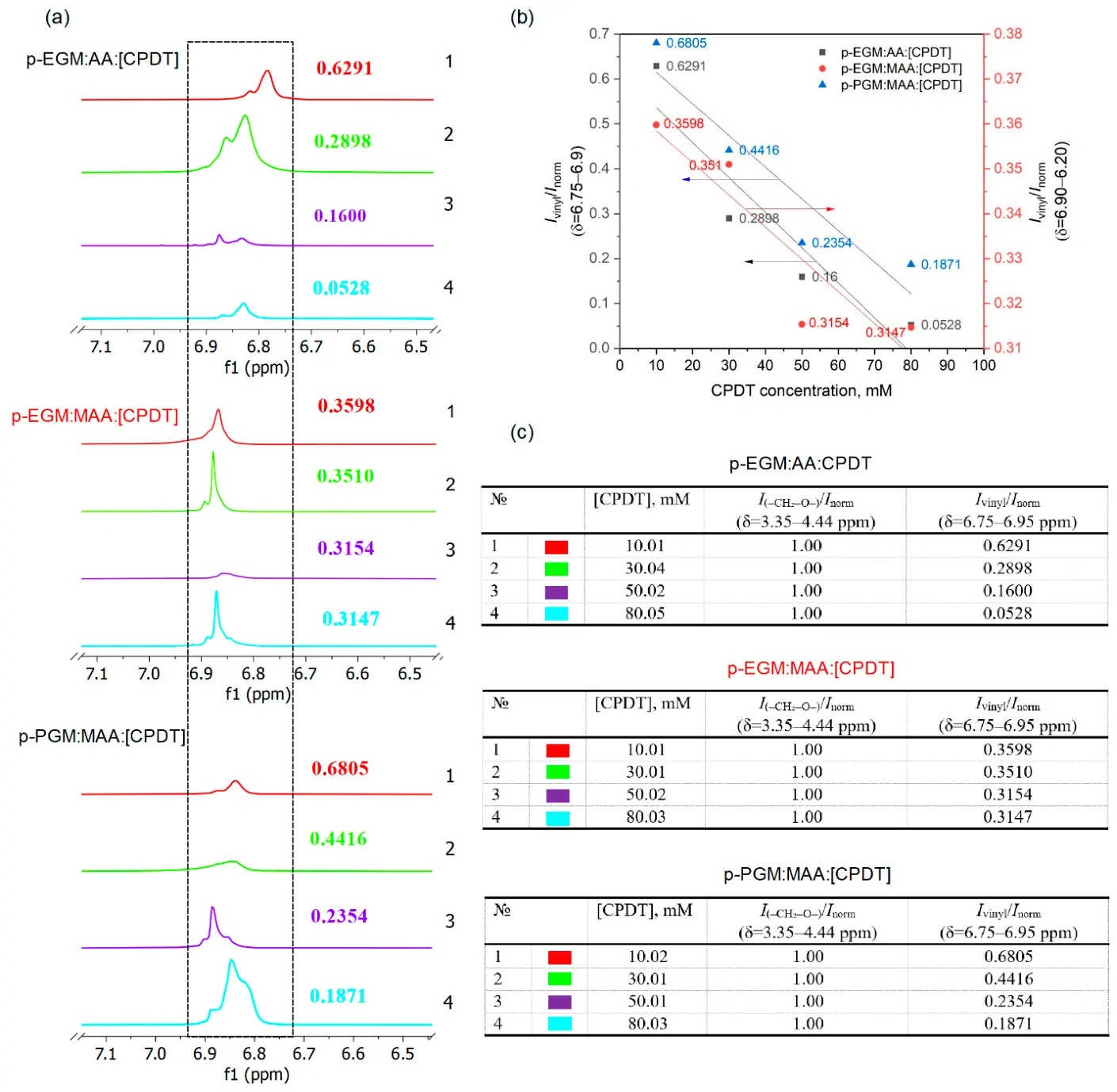
Relative residual maleate vinyl intensity of the investigated copolymer series at different CPDT concentrations: (**a**) representative ^1^H NMR spectral regions corresponding to the residual maleate vinyl protons (*δ* = 6.75–6.95 ppm) for the p-EGM:AA:[CPDT], p-EGM:MAA:[CPDT], and p-PGM:MAA:[CPDT] series; (**b**) dependence of the normalized residual vinyl proton intensity, on the CPDT concentration for the p-EGM:AA:[CPDT], p-EGM:MAA:[CPDT], and p-PGM:MAA:[CPDT] series; and (**c**) corresponding tabulated values of for each copolymer series at different CPDT concentrations. The integrated polyester-backbone region at *δ* = 3.35–4.44 ppm was used as the normalization reference and set to unity. In panel (**b**), black squares, red circles, and blue triangles represent the p-EGM:AA:[CPDT], p-EGM:MAA:[CPDT], and p-PGM:MAA:[CPDT] series, respectively. In panels (**a**,**c**), the colors identify the corresponding CPDT concentrations within each copolymer series.

To evaluate the influence of the CPDT concentration on the relative content of residual maleate double bonds, the normalized integral intensities of the signals in the *δ* = 6.75–6.95 ppm region were compared. The polyester backbone signals in the *δ* = 3.35–4.44 ppm region were used as an internal standard, with their integral intensity normalized to unity (Figure 3c).

Across all investigated copolymer series, increasing the CPDT concentration was accompanied by a decrease in the Relative Vinyl Intensity (Figure 3a,b); however, the magnitude of this decrease depended strongly on the chemical structure of the copolymer.

The most pronounced decrease was observed for the p-EGM:AA:[CPDT] series, in which the Relative Vinyl Intensity decreased from 0.6291 at [CPDT] = 10.01 mM to 0.0528 at [CPDT] = 80.03 mM, corresponding to more than an eleven-fold decrease. For the p-PGM:MAA:[CPDT] series, the corresponding value decreased from 0.6805 to 0.1871, whereas the p-EGM:MAA:[CPDT] series exhibited considerably smaller changes, decreasing only from 0.3598 to 0.3147.

These results show that increasing the CPDT concentration is accompanied by a decrease in the relative NMR contribution of residual maleate vinyl protons in all investigated copolymer series. The p-EGM:AA:[CPDT] series exhibited the highest sensitivity to variations in CPDT concentration, whereas p-PGM:MAA:[CPDT] showed an intermediate response. In contrast, the p-EGM:MAA:[CPDT] copolymers displayed the smallest variation in Relative Vinyl Intensity.

Overall, increasing the CPDT concentration was associated with a reduction in the normalized residual vinyl signal. Combined with the H NMR and H–H COSY data, these changes indicate differences in the extent of residual unsaturation among the investigated copolymer series. A more detailed comparison of these spectral changes is presented in the following section using additional semi-quantitative NMR descriptors.

#### 2.2.4. Semi-Quantitative NMR Analysis of Structural Evolution

Because the investigated RAFT copolymers comprise soluble branched macromolecules together with partially cross-linked polyester domains, direct quantitative determination of the degree of branching using conventional approaches such as the Frey method is not feasible [24]. Unlike well-defined hyperbranched polymers, in which dendritic, linear, and terminal units can be distinguished spectroscopically, the present copolymer systems exhibit extensive signal overlap arising from multiple structural fragments. Therefore, the H NMR integral ratios discussed below are used exclusively as semi-quantitative spectral descriptors for comparing changes in selected proton environments among the investigated samples [14,25,26]. They are not interpreted as direct quantitative measures of branching degree, end-group concentration, or individual structural-unit content.

In addition to the Relative Vinyl Intensity (*RVI*) discussed above, two semi-quantitative descriptors, the CPDT-associated methyl intensity (MI*) and the normalized aliphatic intensity (AI*), were calculated according to Equations (1) and (2):

(1) $${MI}^{*}=\frac{I_{0.75-0.90}}{I_{3.35-4.44}},$$

(2) $${AI}^{*}=\frac{I_{0.90-1.30}}{I_{3.35-4.44}}$$

where $I_{0.75-0.90}$ and $I_{0.90-1.30}$ are the integral intensities of the corresponding spectral regions, and *I*_norm_ is the integral intensity of the polyester reference region at *δ* = 3.35–4.44 ppm.

The polyester backbone signals in the *δ* = 3.35–4.44 ppm region were used as the normalization reference (*I*_norm_ = 1.00). The integral $I_{0.75-0.90}$ represents proton environments assigned predominantly to terminal methyl groups associated with CPDT-derived moieties. Accordingly, MI* is used as a semi-quantitative descriptor of the relative contribution of these CPDT-associated methyl signals and not as a quantitative measure of the RAFT end-group concentration. The calculated $MI^{*}$ values are summarized in Table 1.

The *δ* = 0.90–1.30 ppm region contains strongly overlapping contributions from polymer-derived aliphatic proton environments and the dodecyl alkyl moiety of CPDT. Consequently, AI* represents the normalized overall aliphatic spectral contribution and cannot be assigned specifically to polymer-backbone branching. In particular, variations in AI* with CPDT concentration may reflect changes in both polymer-derived and CPDT-derived contributions. Therefore, no direct quantitative relationship between AI* and the degree of branching is inferred in the present study. 

The CPDT-associated methyl intensity (MI*), calculated as the normalized integral intensity of the *δ* = 0.75–0.90 ppm region relative to the polyester reference region at *δ* = 3.35–4.44 ppm, was used as a semi-quantitative spectral descriptor of the relative contribution of proton environments assigned predominantly to terminal methyl groups associated with CPDT-derived moieties. Across the investigated copolymer series, variations in CPDT concentration were accompanied by changes in MI*; however, the magnitude and direction of these changes depended on the chemical composition of the copolymer.

For the p-EGM:AA:[CPDT] series, MI* decreased from 0.0258 at 10.01 mM CPDT to 0.0137 at 50.01 mM, followed by a slight increase to 0.0163 at 80.03 mM. The p-EGM:MAA:[CPDT] series exhibited relatively small MI* values, ranging from 0.0017 to 0.0075. For the p-PGM:MAA:[CPDT] series, MI* remained nearly unchanged between 10 and 30 mM (0.0056–0.0053), increased to 0.0641 at 50.01 mM, and subsequently decreased to 0.0083 at 80.03 mM.

The variation in MI* with CPDT concentration did not directly parallel the trend observed for the Relative Vinyl Intensity (Figure 3), indicating that the normalized residual vinyl signal and the CPDT-associated methyl signal exhibited distinct spectral responses across the investigated copolymer series. Importantly, MI* is used here only as a semi-quantitative descriptor of the relative contribution of the selected CPDT-associated methyl proton region and should not be interpreted as a quantitative measure of RAFT end-group concentration.

The different MI* profiles observed among the three copolymer series further indicate that the relative contribution of the selected CPDT-associated proton environment depends on both CPDT concentration and copolymer composition. The corresponding values of the normalized aliphatic intensity (AI*) are summarized in Table 2.

For the p-EGM:AA:[CPDT] series, the normalized aliphatic intensity (AI*) decreased from 1.5233 to 0.4377 as the CPDT concentration increased from 10.01 to 50.01 mM, followed by an increase to 1.1300 at 80.03 mM. In contrast, the p-EGM:MAA:[CPDT] copolymers exhibited a considerably narrower range of AI* values (0.1237–0.2934). The p-PGM:MAA:[CPDT] series showed a gradual increase in AI*, reaching a maximum of 0.6394 at 50.01 mM, followed by a decrease to 0.4568 at 80.03 mM.

The different AI* profiles observed among the three copolymer series indicate that the relative contribution of proton environments within the *δ* = 0.90–1.30 ppm region varies with both CPDT concentration and copolymer composition. However, because this spectral region contains strongly overlapping contributions from polymer-derived aliphatic proton environments and the dodecyl alkyl moiety of CPDT, these variations cannot be assigned uniquely to changes in polymer-backbone branching. Accordingly, AI* is interpreted here exclusively as a semi-quantitative descriptor of the overall normalized aliphatic spectral contribution and not as a quantitative measure of the degree of branching.

In particular, changes in AI* with increasing CPDT concentration may reflect simultaneous variations in polymer-derived aliphatic environments and CPDT-derived alkyl contributions. Therefore, no direct relationship between an increase or decrease in AI* and a corresponding increase or decrease in polymer-backbone branching is inferred from these data. The differences observed among the p-EGM:AA:[CPDT], p-EGM:MAA:[CPDT], and p-PGM:MAA:[CPDT] series instead demonstrate composition-dependent changes in the overall aliphatic H NMR spectral contribution.

A combined analysis of the Relative Vinyl Intensity (*RVI*), CPDT-associated methyl intensity (MI*), and normalized aliphatic intensity (AI*) shows that variation in CPDT concentration is accompanied by changes in the relative contributions of residual vinyl, CPDT-associated methyl, and overlapping aliphatic proton environments. The generally lower *RV I* values observed at higher CPDT concentrations reflect a reduction in the normalized residual maleate vinyl signal, although the magnitude and monotonicity of this change depended on the copolymer composition. Taken together, these results provide evidence of changes in the local chemical environments accompanying RAFT copolymerization; however, the semi-quantitative NMR descriptors are not considered direct quantitative measures of macromolecular branching. Accordingly, conclusions regarding RAFT-mediated structural organization are based on the combined interpretation of the NMR results and the GPC/SEC characteristics of the soluble copolymer fraction.

### 2.3. Influence of the Acid Monomer on the ^1^H NMR Spectra

#### Spectral Comparison of AA and MAA Copolymers

**Figure 4:**
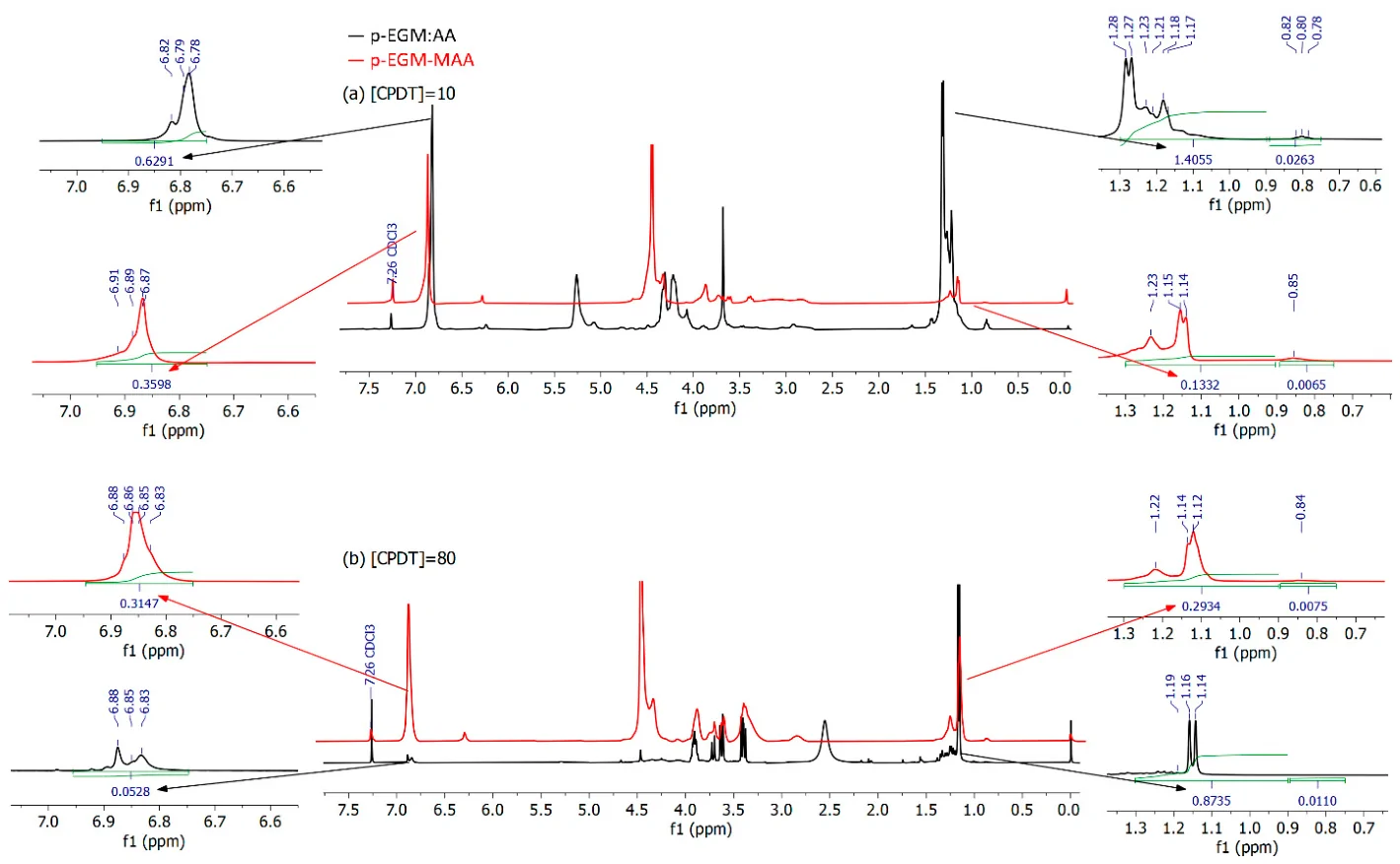
Comparison of the ^1^H NMR spectra of p-EGM:AA:[CPDT] (black) and p-EGM:MAA:[CPDT] (red) copolymers synthesized at (**a**) 10.02 and (**b**) 80.03 mM CPDT. Expanded views of the residual vinyl (*δ* = 6.95–6.75 ppm) and aliphatic (*δ* = 1.30–0.95 ppm) regions are included for comparison.

To evaluate the influence of the acidic comonomer on the structure of the synthesized copolymers, the ^1^H NMR spectra of the p-EGM:AA:[CPDT] and p-EGM:MAA:[CPDT] series synthesized at CPDT concentrations of 10.01 and 80.03 mM were compared (Figure 4). Because both series share the same unsaturated polyester matrix and differ in the acidic comonomer, this comparison provides insight into the influence of acrylic acid and methacrylic acid on the spectral characteristics of the resulting copolymers.

The spectra retain the principal resonance regions characteristic of the investigated copolymer systems over the studied CPDT concentration range. However, variations in the positions, relative intensities, line shapes, and apparent splitting patterns of several resonances are observed as the CPDT concentration changes. These differences indicate changes in the local proton environments and therefore should not be interpreted as spectroscopically invariant structures. At the same time, the absence of a fundamentally new set of resonances suggests that the principal chemical functionalities of the copolymer systems are retained. This observation suggests that no major change in the principal chemical functionalities occurs within either series. The most apparent differences between the spectra are instead observed in the relative integral intensities of selected spectral regions.

The residual vinyl region at *δ* = 6.75–6.95 ppm (Figure 3, enlarged insets) is particularly sensitive to the nature of the acidic comonomer. In both series, increasing the CPDT concentration is accompanied by a decrease in the normalized integral intensity of this region, consistent with the corresponding decrease in Relative Vinyl Intensity (*RVI*). This decrease is considerably more pronounced for the acrylic acid-based p-EGM:AA:[CPDT] series than for the methacrylic acid-based p-EGM:MAA:[CPDT] series, indicating a stronger CPDT-concentration-dependent change in the residual maleate vinyl signal for the AA-containing system.

Differences are also observed in the aliphatic region at *δ* = 0.95–1.30 ppm (Figure 4, enlarged insets). The p-EGM:MAA:[CPDT] spectra exhibit higher relative signal intensities in parts of this region than the corresponding p-EGM:AA:[CPDT] spectra. The additional -methyl group of methacrylic acid-derived units is expected to contribute to this difference. However, because this spectral region also contains overlapping contributions from other polymer-derived aliphatic proton environments and the dodecyl alkyl moiety of CPDT, the observed intensity differences cannot be attributed exclusively to the methacrylic acid -methyl group.

Overall, the comparison demonstrates that the nature of the acidic comonomer influences the ^1^H NMR spectral response of the investigated RAFT copolymers. The different profiles observed for *RVI*, CPDT-associated methyl intensity (MI*), and normalized aliphatic intensity (AI*) indicate composition-dependent changes in the relative contributions of residual vinyl, CPDT-associated methyl, and overlapping aliphatic proton environments. These semi-quantitative descriptors are therefore used to compare spectral changes between the AA- and MAA-containing systems and are not interpreted as direct quantitative measures of macromolecular branching.

### 2.4. Effect of the Unsaturated Polyester Resin Structure

**Figure 5:**
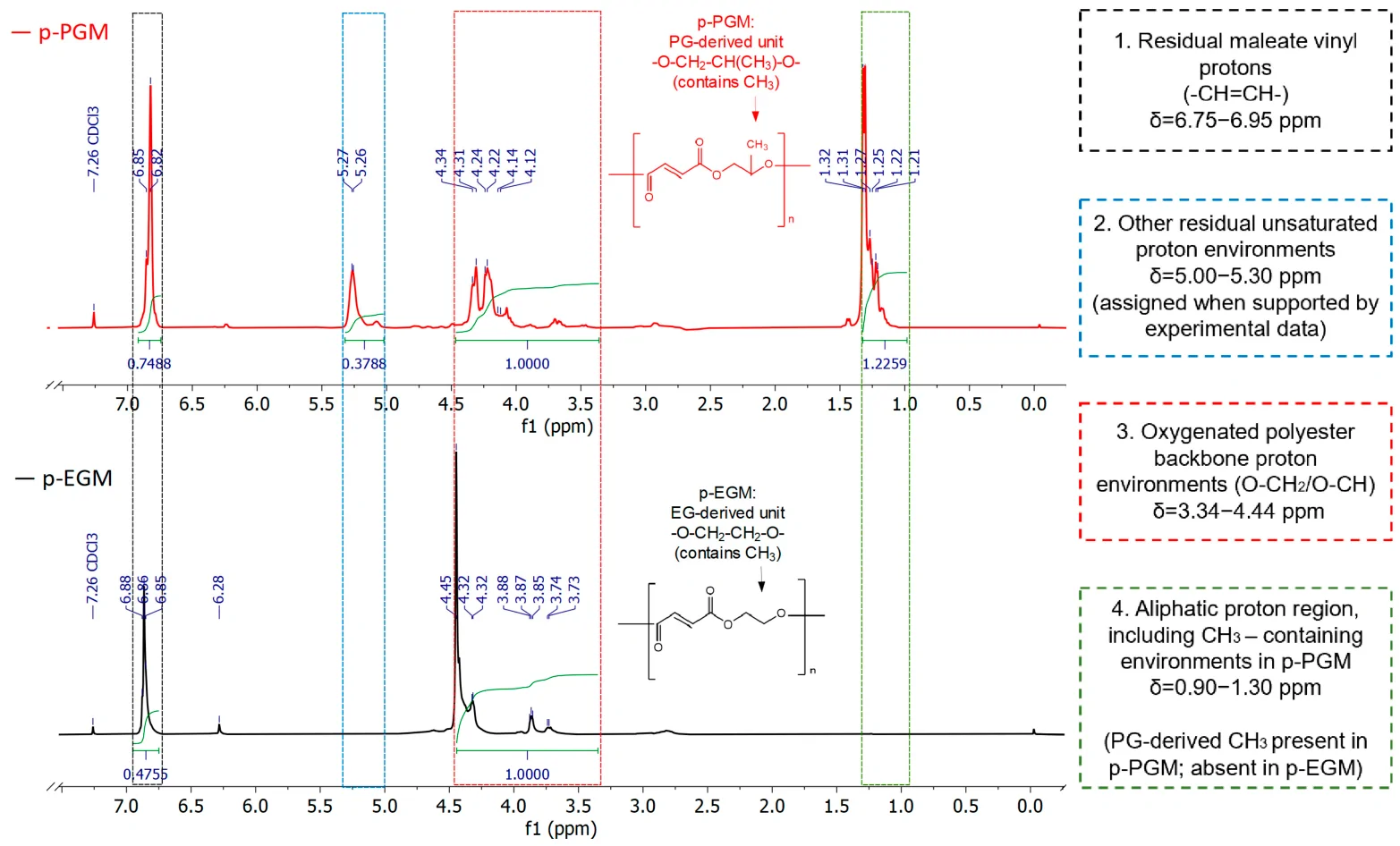
Comparison of the ^1^H NMR spectra of the parent p-PGM and p-EGM polyester resins. The characteristic regions of the residual maleate vinyl protons (*δ* = 6.95–6.75 ppm), other residual unsaturated proton environments (*δ* = 5.30–5.00 ppm), oxygenated polyester-backbone proton environments (*δ* = 4.44–3.34 ppm), and the aliphatic proton region (*δ* = 1.30–0.90 ppm), including CH_3_-containing environments in p-PGM, are highlighted. The structural insets illustrate the characteristic glycol-derived backbone environments of p-EGM [–O–CH_2_–CH_2_–O–] and p-PGM [–O–CH_2_–CH(CH_3_)–O–].

To evaluate the influence of the polyester backbone structure on the spectral characteristics of the synthesized materials, the ^1^H NMR spectra of the parent polyester resins, p-PGM and p-EGM, were compared (Figure 5). Because both resins contain no acidic comonomer and differ only in the chemical structure of their diol-derived units, this comparison enables direct evaluation of the influence of the polyester matrix on the spectral characteristics of the corresponding prepolymers.

Both parent resins exhibit the characteristic resonances of the maleate-containing polyester backbone, while their aliphatic spectral regions differ because of the different glycol-derived units. In particular, p-PGM contains the propylene-glycol-derived –O–CH_2_–CH(CH_3_)–O– environment, whereas p-EGM contains the ethylene-glycol-derived –O–CH_2_–CH_2_–O– unit and therefore lacks the corresponding backbone methyl group. The chemical shifts in the principal resonances remain virtually identical (Figure 5), indicating that the fundamental chemical structure of the principal structural fragments is preserved. The primary differences between the spectra are reflected in the relative integral intensities of characteristic spectral regions rather than in their chemical shifts.

The most noticeable differences are observed in the polyester backbone region (*δ* = 3.34–4.44 ppm) and in the aliphatic region (*δ* = 0.90–1.30 ppm) (Figure 5). These differences are consistent with the different chemical structures of the p-PGM and p-EGM polyester matrices, particularly the presence of the additional methyl substituent in the propylene glycol units of p-PGM.

Changing the chemical structure of the polyester backbone does not generate new resonances or produce significant changes in the chemical shifts in the characteristic signals. Instead, it results in different relative integral intensities within the characteristic ^1^H NMR regions. Consequently, the parent p-PGM and p-EGM polyester matrices exhibit distinct spectral characteristics that influence the spectra of their corresponding RAFT copolymers.

To determine how these differences between the parent polyester matrices are reflected after RAFT copolymerization, the ^1^H NMR spectra of the corresponding p-PGM:MAA:[CPDT] and p-EGM:MAA:[CPDT] copolymers are compared in the following section.

#### Spectral Comparison of p-PGM:MAA:[CPDT] and p-EGM:MAA:[CPDT]

**Figure 6:**
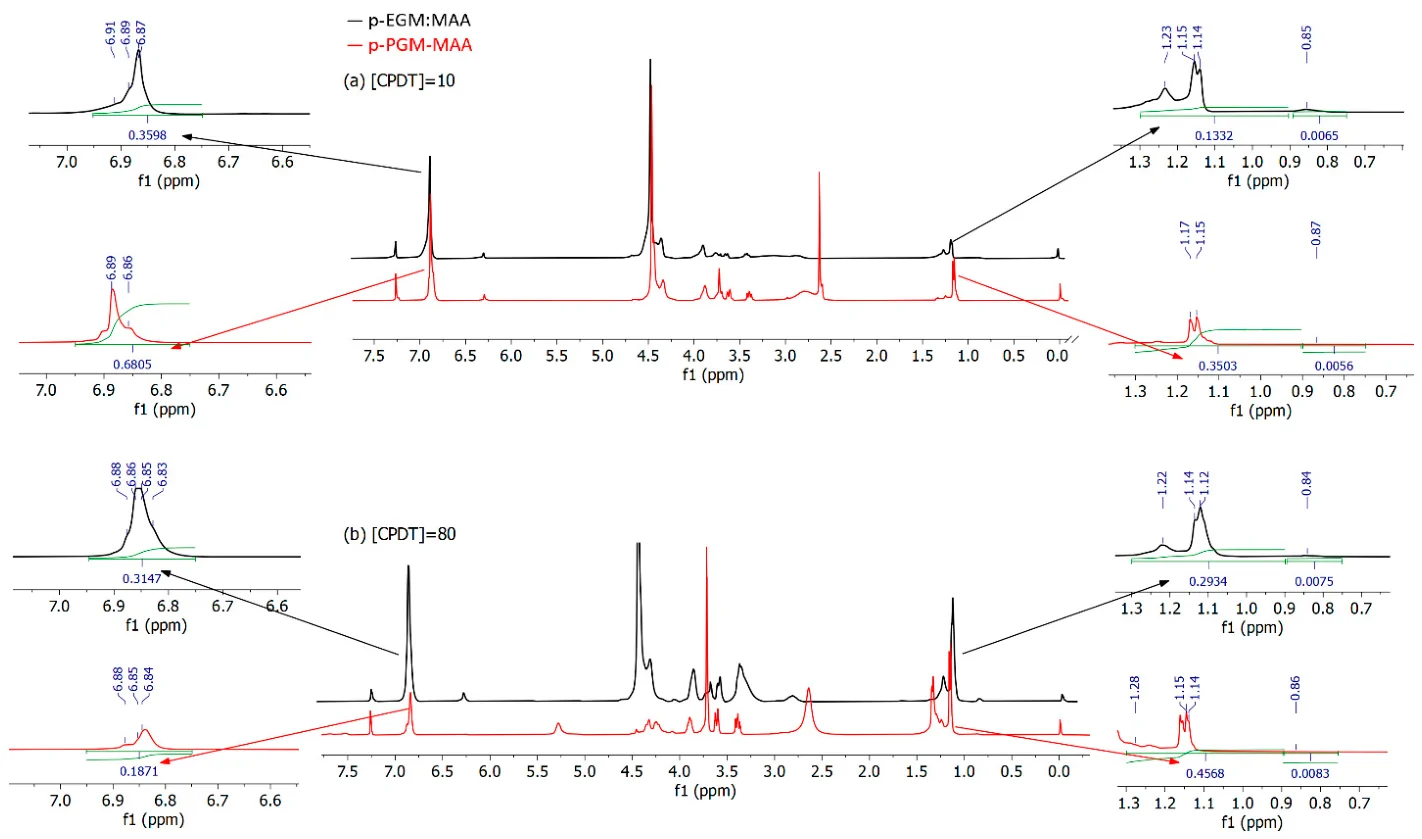
Comparison of the ^1^H NMR spectra of p-EGM:MAA:[CPDT] (black) and p-PGM:MAA:[CPDT] (red) copolymers synthesized at (**a**) 10.01 and (**b**) 80.03 mM CPDT. Enlarged views of the residual vinyl (*δ* = 6.95–6.75 ppm) and aliphatic (*δ* = 1.30–0.90 ppm) regions are shown for comparison.

The preceding analysis demonstrated that the nature of the acidic comonomer is an important factor influencing the structural evolution of the investigated RAFT copolymers. To further evaluate the contribution of the second major structural parameter—the chemical structure of the polyester matrix—the ^1^H NMR spectra of the p-EGM:MAA:[CPDT] and p-PGM:MAA:[CPDT] series synthesized at identical CPDT concentrations were compared (Figure 6).

Figure 6 presents the ^1^H NMR spectra of the p-EGM:MAA:[CPDT] and p-PGM:MAA:[CPDT] copolymers synthesized at CPDT concentrations of 10.01 and 80.03 mM. In both series, the characteristic resonances corresponding to the residual maleate double bonds (*δ* = 6.75–6.95 ppm), the polyester backbone (*δ* = 3.34–4.44 ppm), and the aliphatic fragments (*δ* = 0.90–1.30 ppm) are preserved with increasing CPDT concentration. Furthermore, the chemical shifts in these characteristic resonances remain virtually unchanged both with increasing CPDT concentration and when the polyester matrix is changed from p-EGM to p-PGM. This observation indicates that the fundamental chemical structure of the principal structural fragments is maintained throughout the RAFT copolymerization process.

Consequently, changing the chemical structure of the polyester matrix does not introduce new resonances or produce significant changes in the chemical shifts in the characteristic resonances. Instead, it results in distinct changes in the relative integral intensities of characteristic ^1^H NMR regions. These findings indicate that both the chemical structure of the polyester matrix and the nature of the acidic comonomer strongly influence the spectral characteristics and macromolecular architecture of the resulting RAFT copolymers.

### 2.5. UV–Vis Characterization of p-PGM:MAA:[CPDT] Copolymers

**Figure 7:**
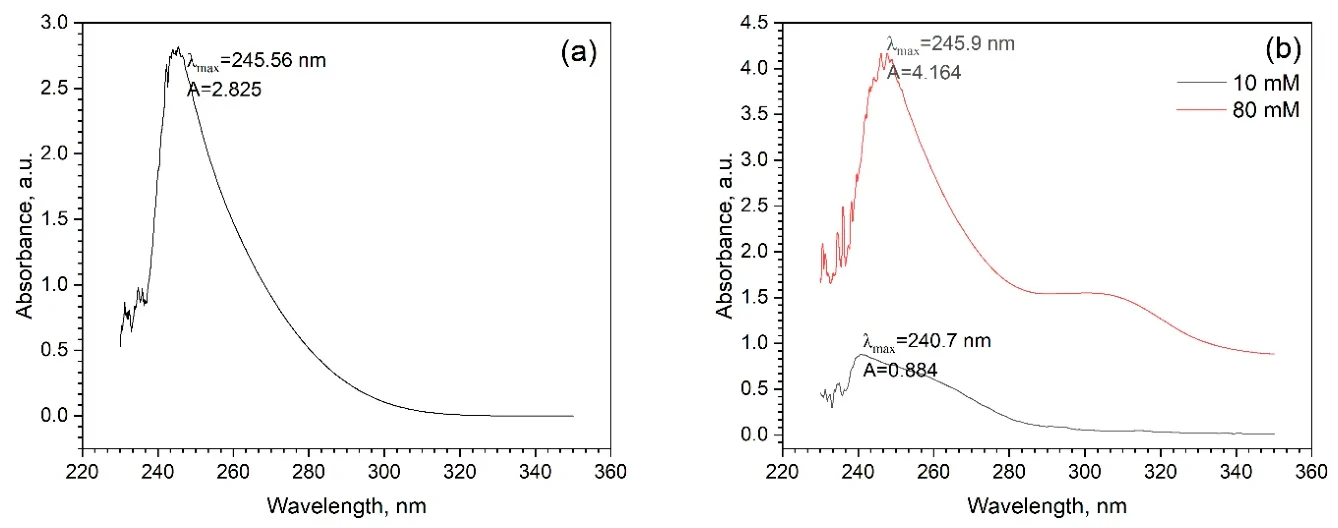
UV–Vis absorption spectra of (**a**) the initial p-PGM resin and (**b**) p-PGM:MAA:[CPDT] copolymers synthesized at CPDT concentrations of 10.01 and 80.03 mM.

To evaluate the influence of CPDT concentration on the electronic absorption characteristics of the investigated copolymers, the p-PGM:MAA:[CPDT] series synthesized at CPDT concentrations of 10.01 and 80.03 mM was selected as a model system. For comparison, the UV–Vis spectrum of the parent p-PGM polyester resin was also recorded (Figure 7).

As shown in Figure 7, the parent p-PGM resin exhibits an intense absorption band with a maximum at *λ*_max_ = 245.6 nm. Following RAFT copolymerization, the principal absorption band remains in the same spectral region, with *λ*_max_ values of 240.7 nm for the 10.01 mM CPDT sample and 245.9 nm for the 80.03 mM sample. The absence of substantial changes in the absorption maxima indicates that the fundamental electronic transitions responsible for this absorption band remain essentially unchanged.

Increasing the CPDT concentration is accompanied by a substantial increase in the absorption intensity (*A* = 0.884–4.164) together with more pronounced absorption in the 250–330 nm region. These changes indicate differences in the relative contributions of the chromophoric groups involved in the electronic transitions while preserving the overall electronic structure of the copolymer chromophoric system.

These observations are consistent with the ^1^H NMR results, which likewise show that increasing the CPDT concentration primarily affects the relative intensities of the characteristic signals without causing significant changes in their chemical shifts. Thus, the UV–Vis and ^1^H NMR data provide complementary information, indicating that variation in CPDT concentration modifies the relative spectral characteristics of the investigated copolymers while preserving the fundamental chemical structure of their principal structural fragments.

### 2.6. Molecular-Weight Characterization of p-EGM:MAA:[CPDT] and p-PGM:MAA:[CPDT] Copolymers by GPC

**Figure 8:**
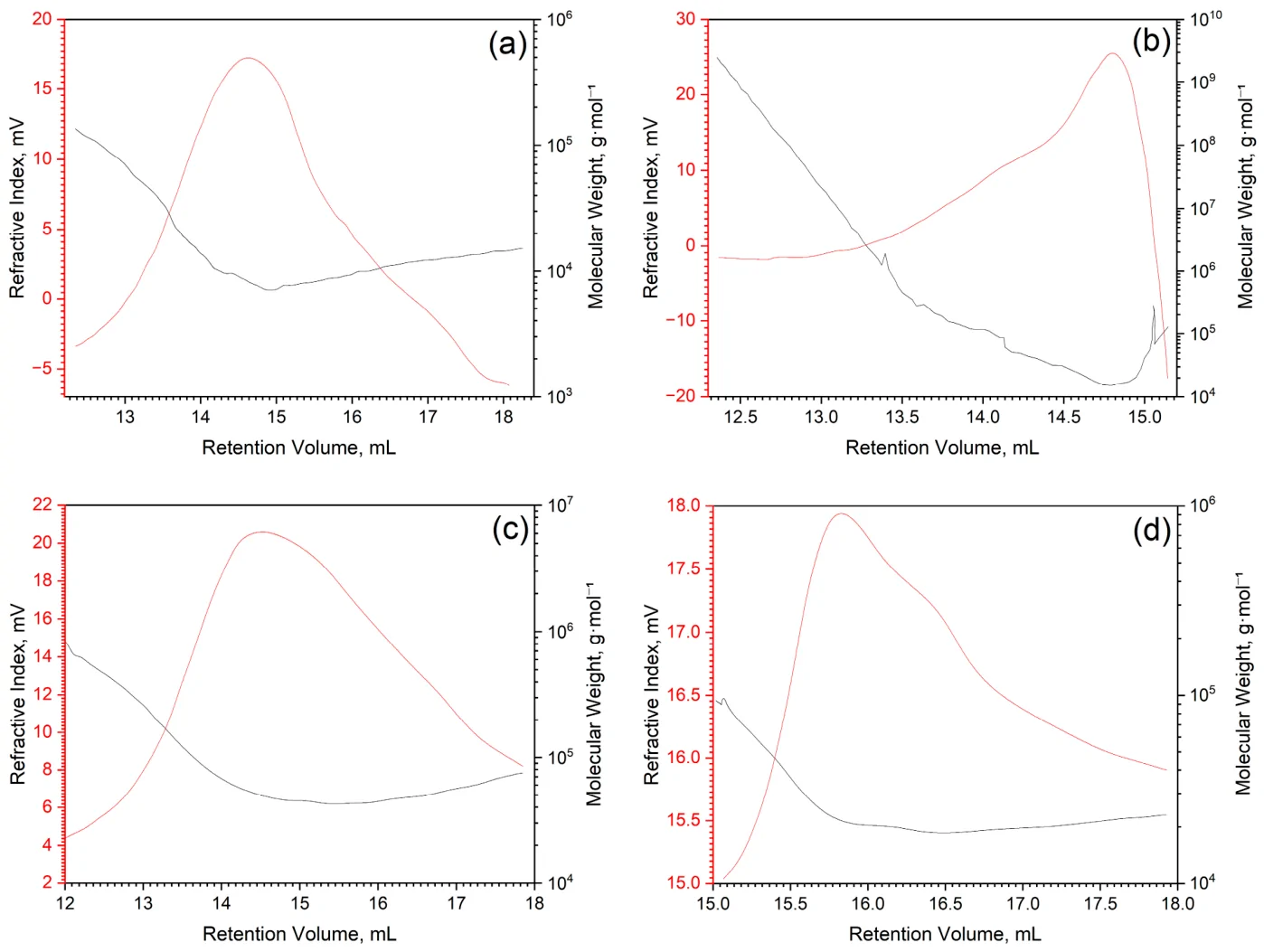
GPC chromatograms of (**a**,**c**) p-EGM:MAA:[CPDT] and (**b**,**d**) p-PGM:MAA:[CPDT], showing the refractive-index detector response (left axis) and the point-by-point molecular-weight trace generated by the OmniSEC software (right logarithmic axis). The number-average, weight-average, and z-average molecular weights and dispersities calculated for the integrated polymer peaks are summarized separately in Table 3.

To complement the spectroscopic characterization, the molecular-weight characteristics of the investigated RAFT copolymers were evaluated by gel permeation chromatography (GPC). The analysis was performed for the p-EGM:MAA:[CPDT] and p-PGM:MAA:[CPDT] series synthesized at CPDT concentrations of 10.01 and 80.03 mM. Figure 8 presents the resulting GPC chromatograms, including the refractive index (RI) detector traces and the corresponding molecular-weight distribution curves.

Both copolymer series exhibit unimodal molecular-weight distributions corresponding to the soluble fraction of the synthesized materials. Increasing the CPDT concentration from 10.01 to 80.03 mM results in changes in both the chromatographic profile and the elution volume, indicating systematic changes in the molecular-weight characteristics of the copolymers. The calculated values of the number-average molecular weight (*M_n_*), weight-average molecular weight (*M_W_*), z-average molecular weight (*M_Z_*), and dispersity (*Đ*) are summarized in Table 3.

At a CPDT concentration of 10.01 mM, the p-PGM:MAA:[CPDT] copolymer exhibits higher *M_n_*, *M_W_*, and *M_Z_* values than the corresponding p-EGM:MAA:[CPDT] copolymer. Furthermore, the PGM-based copolymer displays a broader molecular-weight distribution, with a higher dispersity (*Đ* = 2.10) than the EGM-based copolymer (*Đ* = 1.27).

The systematically higher molecular-weight values observed for the p-PGM:MAA:[CPDT] soluble fraction cannot be attributed solely to the molecular weight of the parent polyester resin. The parent p-PGM resin exhibits an *M_n_* of 1.213 × 10^3^ g mol^−1^, whereas the parent p-EGM resin exhibits an *M_n_* of 0.959 × 10^3^ g mol^−1^. Thus, the initial *M_n_* of p-PGM is approximately 26% higher than that of p-EGM, which is substantially smaller than the approximately twofold difference observed between the corresponding RAFT copolymer soluble fractions. Therefore, the initial molecular-weight difference contributes to, but does not fully account for, the observed GPC results.

Upon increasing the CPDT concentration to 80.03 mM, both copolymer series exhibit changes in molecular weight and dispersity, demonstrating that the RAFT agent concentration influences the molecular-weight distribution of the soluble copolymer fraction. However, the magnitude of these changes differs between the two polyester matrices, indicating that the response to increasing CPDT concentration depends on the chemical structure of the polyester prepolymer.

Differences in the molecular-weight development of the two polyester systems may also reflect matrix-dependent differences in monomer incorporation and in the effective balance between propagation and CPDT-mediated chain transfer. However, because monomer conversion and chain-transfer efficiency were not independently quantified for the p-EGM- and p-PGM-based systems, these factors cannot be assigned as definitive causes of the observed *M_n_* difference. In particular, the isolated branched polymer yield discussed below should not be interpreted as equivalent to monomer conversion.

The heterogeneous nature of the polymerization must also be considered. Gravimetric analysis demonstrated that the recovered products contain both soluble and insoluble fractions and that their relative proportions vary strongly with CPDT concentration. GPC/SEC therefore characterizes only the isolated soluble fraction rather than the entire reaction product. Consequently, differences in branching and network/gel formation may selectively determine which macromolecular populations remain soluble and are accessible to GPC analysis [14,15]. This limitation is particularly important at low CPDT concentration, where the soluble fraction represents only a small proportion of the recovered product.

The chromatographic interpretation also requires consideration of differences in solution conformation and hydrodynamic behavior. SEC separation is governed by hydrodynamic size, and branched macromolecules may adopt more compact solution conformations than linear macromolecules of comparable molecular weight [20,21]. In the present study, molecular-weight calculations were based on the multidetector response, including RI and light-scattering data, rather than solely on a conventional retention-volume calibration curve. Nevertheless, the light-scattering detector was calibrated using a narrow-molecular-weight-distribution linear polystyrene standard. Although the molecular-weight calculations were based on multidetector RI and light-scattering responses rather than solely on conventional retention-volume calibration against linear standards, potential systematic uncertainties associated with calibration and differences in solution conformation between the linear calibration standard and the branched copolymer fractions cannot be completely excluded. Accordingly, the approximately twofold difference in *M_n_* should not be interpreted as direct evidence that the p-PGM-derived chains are intrinsically twice as long.

Overall, the GPC results complement the ^1^H NMR, ^1^H–^1^H COSY, and UV–Vis data by demonstrating composition-dependent differences in the molecular-weight characteristics of the soluble copolymer fractions. The higher *M_n_* values observed for p-PGM:MAA:[CPDT] relative to p-EGM:MAA:[CPDT] cannot be assigned to a single mechanistic factor. Rather, they are interpreted as potentially reflecting the combined influence of the initial polyester molecular weight, matrix-dependent polymerization and effective chain-transfer behavior, branching and gel formation, selective partitioning into the soluble fraction, and differences in solution conformation and chromatographic response. Accordingly, the GPC-derived molecular weights are interpreted as characteristics of the soluble copolymer fractions and not as direct measures of the molecular weight of the entire heterogeneous reaction product.

**Table 3:** Molecular-Weight Characteristics of the Investigated RAFT Copolymers Synthesized at CPDT Concentrations of 10.01 and 80.03 mM.

Sample	*M_n_*, 10^3^ g·mol^−1^	*M_w_*, 10^3^, g·mol^−1^	*M_z_*, 10^3^, g·mol^−1^	*Đ (M_w_*/*M_n_)*
10.01 mM				
p-EGM:MAA:[CPDT]	9.680	12.235	20.607	1.27
p-PGM:MAA:[CPDT]	23.422	49.369	76.168	2.10
80.03 mM				
p-EGM:MAA:[CPDT]	8.954	10.884	27.887	1.22
p-PGM:MAA:[CPDT]	18.635	25.574	59.025	1.37

**Figure 9:**
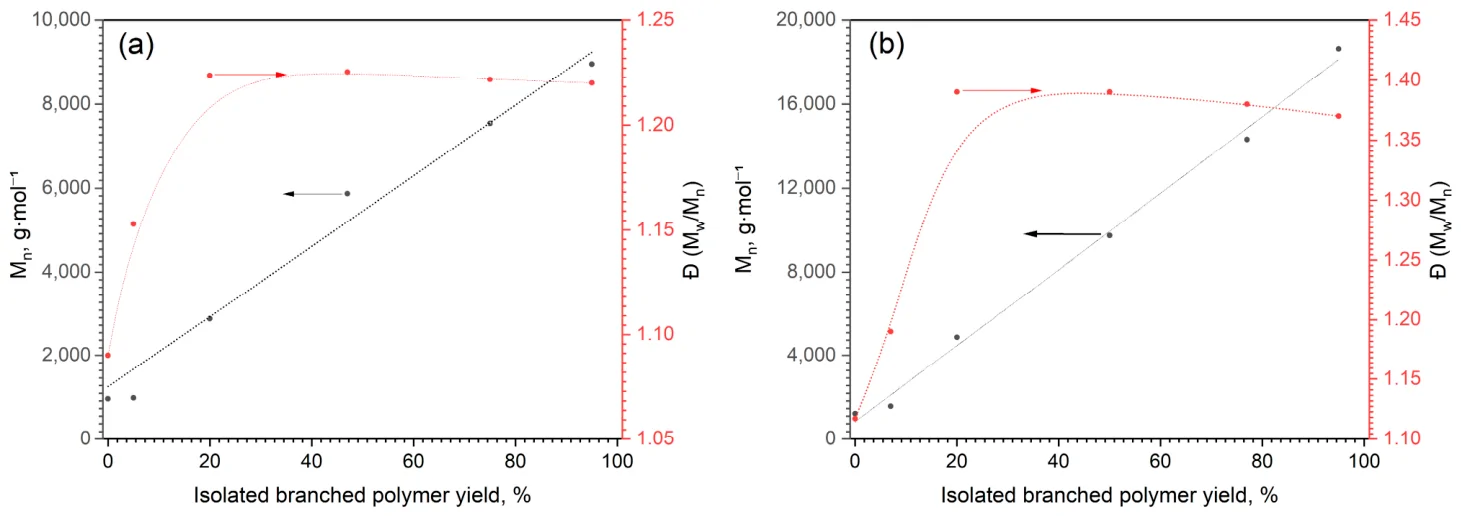
Evolution of the molecular-weight characteristics as a function of isolated branched polymer yield for RAFT copolymers synthesized at [CPDT]_0_ ≈ 80.03 mM and [BP]_0_ ≈ 6.58 mM ([CPDT]_0_/[BP]_0_ = 12.16). Experimental number-average molecular weight (*M*n, left axis) and dispersity (Đ = *M*w/*M*n, right axis) are shown for (**a**) p-EGM:MAA:[CPDT] and (**b**) p-PGM:MAA:[CPDT]. The data points at 0% isolated branched polymer yield correspond to the experimentally measured molecular-weight characteristics of the respective parent p-EGM and p-PGM resins and are included as baseline values for comparison with the isolated branched copolymer fractions. The final molecular-weight characteristics are consistent with the corresponding values reported in Table 3. Lines are shown only as guides to the eye.

The evolution of the molecular-weight characteristics with increasing isolated branched polymer yield was evaluated at a constant CPDT concentration of 80.03 mM (Figure 9). For the p-EGM:MAA:[CPDT] system, the number-average molecular weight (*M_n_*) increased from the parent-resin baseline value of 0.959 × 10^3^ g mol^−1^ at 0% isolated branched polymer yield to 8.954 × 10^3^ g mol^−1^ at the highest investigated yield. The dispersity initially increased relative to that of the parent p-EGM resin and subsequently approached approximately 1.22 at higher isolated branched polymer yields. A similar increase in *M_n_* was observed for the p-PGM:MAA:[CPDT] system, starting from the parent p-PGM baseline (*M_n_* = 1.213 × 10^3^ g mol^−1^) and reaching 18.635 × 10^3^ g mol^−1^ at the highest investigated yield. The corresponding dispersity values likewise approached a relatively stable range at higher yields. These trends describe the molecular-weight evolution of the isolated soluble copolymer fractions and should not be interpreted as direct monomer-conversion profiles.

The GPC results are consistent with the ^1^H NMR data. Whereas ^1^H NMR spectroscopy reveals structural differences between the investigated copolymer series, GPC demonstrates that these differences are accompanied by corresponding changes in the molecular-weight characteristics of the soluble fraction. Together, these results indicate that the chemical structure of the polyester matrix influences both the spectral characteristics and the molecular-weight characteristics of the resulting RAFT copolymers.

## 3. Materials and Methods

### 3.1. Materials

Propylene glycol, maleic anhydride, acrylic acid (AA), methacrylic acid (MAA), benzoyl peroxide (BP), zinc chloride, 1,4-dioxane, and the RAFT agent 2-cyano-2-propyl dodecyl trithiocarbonate (CPDT) were purchased from Sigma-Aldrich (Burlington, MA, USA). All reagents were used as received without further purification. In particular, AA and MAA were used without additional purification or inhibitor removal. The same batch of each monomer was used within the corresponding experimental series to maintain consistent polymerization conditions.

### 3.2. Synthesis of Unsaturated Polyester Resins

Poly(ethylene glycol maleate) (p-EGM) and poly(propylene glycol maleate) (p-PGM) were synthesized by polycondensation of maleic anhydride with the corresponding glycol at 423–453 K according to a previously reported procedure [23]. Specifically, p-EGM was synthesized using ethylene glycol, whereas p-PGM was synthesized using propylene glycol. The molecular-weight characteristics of the resulting parent polyester resins were determined by gel permeation chromatography (GPC), and the corresponding number-average molecular weight (*M_n_*), weight-average molecular weight (*M_w_*), and dispersity (*Đ* = *M_w_*/*M_n_*) values are summarized in Table 4.

### 3.3. Radical Copolymerization of Unsaturated Polyester Resins with Acrylic and Methacrylic Acids

Radical copolymerization was carried out for three copolymer systems: p-EGM:AA, p-EGM:MAA, and p-PGM:MAA. The reactions were performed in 1,4-dioxane (1:1 by weight) at 343 K using an equimolar resin-to-monomer ratio (50:50 mol%). Benzoyl peroxide (BP) was used as the radical initiator. In all polymerization experiments, the amount of BP was kept constant at 0.0329 mmol (7.9 mg) in a total reaction volume of 5.0 mL, corresponding to an initial BP concentration of 6.58 mM. Prior to polymerization, the reaction mixtures were degassed for 30 min, sealed in glass ampoules, and maintained at 343 K for 52 h.

### 3.4. RAFT Copolymerization of Unsaturated Polyester Resins with Acrylic and Methacrylic Acids

RAFT copolymerization was carried out for three copolymer systems: p-EGM:AA:[CPDT], p-EGM:MAA:[CPDT], and p-PGM:MAA:[CPDT], under conditions similar to those used for conventional radical copolymerization, but in the presence of different concentrations of the RAFT agent 2-cyano-2-propyl dodecyl trithiocarbonate (CPDT). The BP concentration was maintained constant at 6.58 mM, whereas the initial CPDT concentration was varied from approximately 10 to 80 mM. Accordingly, the [CPDT]_0_/[BP]_0_ molar ratios were approximately 1.52, 4.56, 7.60, and 12.16 (12.17 for [CPDT]_0_ = 80.05 mM), depending on the exact CPDT concentration used. The reaction scheme illustrating the preparation of the investigated RAFT-modified copolymers is presented in Scheme S1 in the Supplementary Materials (Figure S4).

The reaction mixtures were purged with an inert gas, sealed in glass ampoules, and thermostated at 343 K (±0.10 °C) for 52 h. After completion of the reaction, the ampoules were cooled and opened. The cross-linked polymers were separated from the mother liquor by filtration and dried under vacuum at 323–333 K until constant weight. The branched copolymers were subsequently isolated from the mother liquor by precipitation in alcohol [27].

For the kinetic experiments presented in Figure 8, multiple independent glass ampoules with identical initial compositions were prepared in parallel and thermostated at 343 K. At predetermined reaction times, individual ampoules were removed from the thermostat and cooled to terminate the polymerization. Each ampoule was used as a sacrificial sample corresponding to a single kinetic point. The branched polymer fraction was isolated from the mother liquor, purified, and dried under vacuum at 323–333 K to constant weight. The yield of the isolated branched polymer fraction (*Y_b_*) was determined gravimetrically according to Equation (3):

(3) $$Y_{b}\left(\%\right)=\frac{m_{b}}{m_{0}}\cdot 100$$

where *m_b_* is the mass of the isolated, purified, and dried branched polymer fraction and *m*_0_ is the initial total mass of the polymerizable components (unsaturated polyester resin and AA or MAA). The resulting values represent the gravimetric yield of the isolated branched polymer fraction and should not be interpreted as the conversion of either the polyester maleate units or AA/MAA. The molecular-weight characteristics of the corresponding soluble branched polymer fractions were subsequently determined by GPC.

The chemical structures of all synthesized RAFT-modified copolymers and the corresponding initial unsaturated polyester resins were characterized by ^1^H and ^13^C nuclear magnetic resonance (NMR) spectroscopy using a JNM-ECA JEOL 400 spectrometer (JEOL Ltd., Tokyo, Japan), operating at 399.78 and 100.53 MHz for ^1^H and ^13^C nuclei, respectively. CDCl_3_ was used as the solvent, and the chemical shifts were referenced to the residual solvent signals.

Gel permeation/size-exclusion chromatography (GPC/SEC) was used to determine the molecular-weight characteristics of the soluble copolymer fractions. Measurements were performed using a Viscotek 270 multidetector GPC/SEC system (system designation 270-IR) equipped with refractive-index (RI), right-angle light-scattering (RALS), low-angle light-scattering (LALS), and differential viscometric detection. A T6000M column set was used with 1,4-dioxane as the eluent at a flow rate of 1.0 mL min^−1^. The injection volume was 100 μL. The column and detector temperatures were maintained at 35 and 25 °C, respectively. Data acquisition and molecular-weight calculations were performed using OmniSEC software (Build 438). Molecular-weight calculations were based on the multidetector response, including RI and light-scattering data. The light-scattering detector was calibrated using a narrow-molecular-weight-distribution polystyrene standard with a nominal molecular weight of approximately 105 kDa. The number-average molecular weight (*M_n_*), weight-average molecular weight (*M_w_*), z-average molecular weight (*M_z_*), and dispersity (*Đ* = *M_w_*/*M_n_*) were obtained from the processed molecular-weight distributions.

Ultraviolet–visible (UV–Vis) absorption spectra of the copolymer samples were recorded over the wavelength range of 230–350 nm. Polymer solutions were prepared in 1,4-dioxane at a concentration of 1.0 mg mL^−1^ and measured using a quartz cuvette with a 10 mm (1 cm) optical path length. Pure 1,4-dioxane was used as the reference (blank), and solvent baseline correction was performed prior to measurement of the polymer solutions. Identical solution concentrations, cuvette path lengths, and measurement conditions were used for all samples shown in Figure 6.

## 4. Conclusions

This study demonstrates that the structural response of RAFT-modified unsaturated polyester copolymers depends on CPDT concentration, the chemical structure of the polyester matrix, and the nature of the acidic comonomer. The combined ^1^H NMR, ^1^H–^1^H COSY, UV–Vis, and GPC/SEC results reveal composition-dependent changes in residual unsaturation, selected proton environments, and the molecular-weight characteristics of the soluble copolymer fraction.

The principal mechanistic insight supported by these results is that the response to increasing CPDT concentration is not universal across the investigated polyester systems but is strongly dependent on the chemical composition of the reaction system. Thus, the effects associated with CPDT cannot be considered independently of the polyester matrix and acidic comonomer when comparing or designing RAFT-modified unsaturated polyester systems. However, the present data do not establish a quantitative relationship between CPDT concentration and the degree of macromolecular branching.

The semi-quantitative NMR parameters used in this study should therefore be regarded as comparative spectral descriptors rather than direct measures of branching or absolute RAFT end-group concentration. Likewise, the GPC/SEC-derived molecular-weight characteristics describe only the soluble copolymer fraction and should not be extrapolated quantitatively to the entire heterogeneous reaction product without consideration of the insoluble fraction.

Overall, this comparative analysis identifies the polyester matrix and acidic comonomer as important variables governing the structural response of these systems to changes in CPDT concentration. From a practical perspective, the results show that the effect of CPDT concentration cannot be transferred directly from one polyester–comonomer system to another; optimization of RAFT-modified unsaturated polyester formulations should therefore account for both the polyester backbone and the acidic comonomer. These findings provide guidance for selecting and comparing reaction conditions in future studies, while the identified analytical limitations should be considered when relating soluble fraction molecular characteristics to the properties of the heterogeneous bulk material.

## 5. Patents

The methods for preparing the unsaturated polyester resins described in this manuscript, including the use of polypropylene glycol fumarate phthalate as a key component, are protected by a patent [23].

## Figures and Tables

**Table 1:** Semi-quantitative CPDT-associated methyl intensity (MI*) values for the investigated RAFT copolymer series synthesized at different CPDT concentrations.

CPDT, mM	p-EGM:AA:[CPDT]	p-EGM:MAA:[CPDT]	p-PGM:MAA:[CPDT]
10.01	0.0258	0.0017	0.0056
30.01	0.0144	0.0075	0.0053
50.01	0.0137	0.0034	0.0641
80.03	0.0163	0.0065	0.0083

**Table 2:** Normalized aliphatic intensity (AI*) values for the investigated RAFT copolymer series synthesized at different CPDT concentrations.

CPDT, mM	p-EGM:AA:[CPDT]	p-EGM:MAA:[CPDT]	p-PGM:MAA:[CPDT]
10.01	1.5233	0.1422	0.3503
30.01	0.7703	0.2934	0.5045
50.01	0.4377	0.1237	0.6394
80.03	1.1300	0.1332	0.4568

**Table 4:** Molecular-weight characteristics of the parent p-EGM and p-PGM polyester resins determined by GPC.

Parent Resin	*M_n_*, 10^3^ g mol^−1^	*M_w_*, 10^3^ g mol^−1^	*Đ* (*M_w_*/*M_n_*)
p-EGM	0.959	1.041	1.087
p-PGM	1.213	1.355	1.117

## Data Availability

The original contributions presented in this study are included in the article/Supplementary Material. Further inquiries can be directed to the corresponding authors.

## Supplementary Materials

The following supporting information can be downloaded at: https://www.mdpi.com/article/10.3390/molecules31172958/s1, Table S1: Gravimetric mass balance of soluble and insoluble fractions of RAFT-modified copolymers at different CPDT concentrations; Figure S1: ^1^H and ^13^C NMR spectra of p-EGM:AA:[CPDT] copolymers synthesized in the presence of different concentrations of the RAFT agent CPDT: (**a**,**b**) 10 mM; (**c**,**d**) 30 mM; (**e**,**f**) 50 mM; and (**g**,**h**) 80 mM. Panels (**a**,**c**,**e**,**g**) show the ^1^H NMR spectra, whereas panels (**b**,**d**,**f**,**h**) show the ^13^C NMR spectra; Figure S2: ^1^H and ^13^C NMR spectra of p-EGM:MAA:[CPDT] copolymers synthesized in the presence of different concentrations of the RAFT agent CPDT: (**a**,**b**) 10 mM; (**c**,**d**) 30 mM; (**e**,**f**) 50 mM; and (**g**,**h**) 80 mM. Odd-numbered panels correspond to ^1^H NMR spectra, whereas even-numbered panels correspond to ^13^C NMR spectra; Figure S3: ^1^H and ^13^C NMR spectra of p-PGM:MAA:[CPDT] copolymers synthesized in the presence of different concentrations of the RAFT agent CPDT: (**a**,**b**) 10 mM; (**c**,**d**) 30 mM; (**e**,**f**) 50 mM; and (**g**,**h**) 80 mM. Odd-numbered panels correspond to ^1^H NMR spectra, whereas even-numbered panels correspond to ^13^C NMR spectra; Scheme S1: Synthetic route for the preparation of the investigated RAFT-modified unsaturated polyester copolymers p-EGM:AA:[CPDT], p-EGM:MAA:[CPDT], and p-PGM:MAA:[CPDT].

## Abbreviations

The following abbreviations are used in this manuscript:

RAFT	Reversible Addition–Fragmentation Chain Transfer
CPDT	2-Cyano-2-propyl dodecyl trithiocarbonate
UPRs	Unsaturated polyester resins
p-PGM	poly propylene glycol maleate
p-EGM	poly ethylene glycol maleate
AA	Acrylic acid
MAA	Methacrylic acid
NMR	Nuclear magnetic resonance
^1^H NMR	^1^H nuclear magnetic resonance
^1^H–^1^H COSY	^1^H–^1^H correlation spectroscopy
GPC	Gel permeation chromatography
UV–Vis spectroscopy	Ultraviolet–visible absorption spectroscopy
