# Supplementary material for: Structural Evolution of RAFT-Modified Unsaturated Polyester Copolymers: Effects of CPDT Concentration, Acidic Comonomer Structure, and Polyester Matrix Architecture
Source: Molecules. 2026 Aug 24;31(17):2958. doi: 10.3390/molecules31172958 (PMC13566969; doi:10.3390/molecules31172958)
Supplement: Supplementary file 1 [file molecules-31-02958-s001.zip › Table S1.pdf]

Table S1. Gravimetric mass balance of soluble and insoluble fractions of RAFT-modified copolymers at different CPDT concentrations

Copolymer series	CPDT, mM	Insoluble mass, g	Soluble mass, g	Recovery, %	Soluble fraction, %	Insoluble fraction, %
p-EGM:AA:[CPDT]	10.01	3.7892	0.1516	98.52	3.85	96.15
	30.01	3.1628	0.5392	92.55	14.57	85.43
	50.01	2.0016	1.8860	97.19	48.51	51.49
	80.03	0.2428	3.6832	98.15	93.82	6.18
p-EGM:MAA:[CPDT]	10.01	3.7764	0.1556	98.30	3.96	96.04
	30.01	3.1516	0.5404	92.30	14.64	85.36
	50.01	2.0008	1.8964	97.43	48.66	51.34
	80.05	0.2148	3.6984	97.83	94.51	5.49
p-PGM:MAA:[CPDT]	10.02	3.7688	0.1580	98.17	4.02	95.98
	30.01	3.1328	0.5384	91.78	14.67	85.33
	50.01	1.9804	1.8952	96.89	48.90	51.10
	80.03	0.2004	3.6416	96.05	94.78	5.22

Note: The initial mass of polymerizable components was 4.0000 g for all experiments. Recovery was calculated relative to the initial mass, whereas the soluble and insoluble fractions were calculated relative to the total recovered mass.
