# Supplementary material for: Structural Evolution of RAFT-Modified Unsaturated Polyester Copolymers: Effects of CPDT Concentration, Acidic Comonomer Structure, and Polyester Matrix Architecture
Source: Molecules. 2026 Aug 24;31(17):2958. doi: 10.3390/molecules31172958 (PMC13566969; doi:10.3390/molecules31172958)
Supplement: Supplementary file 1 [file molecules-31-02958-s001.zip › Figure S1.pdf]

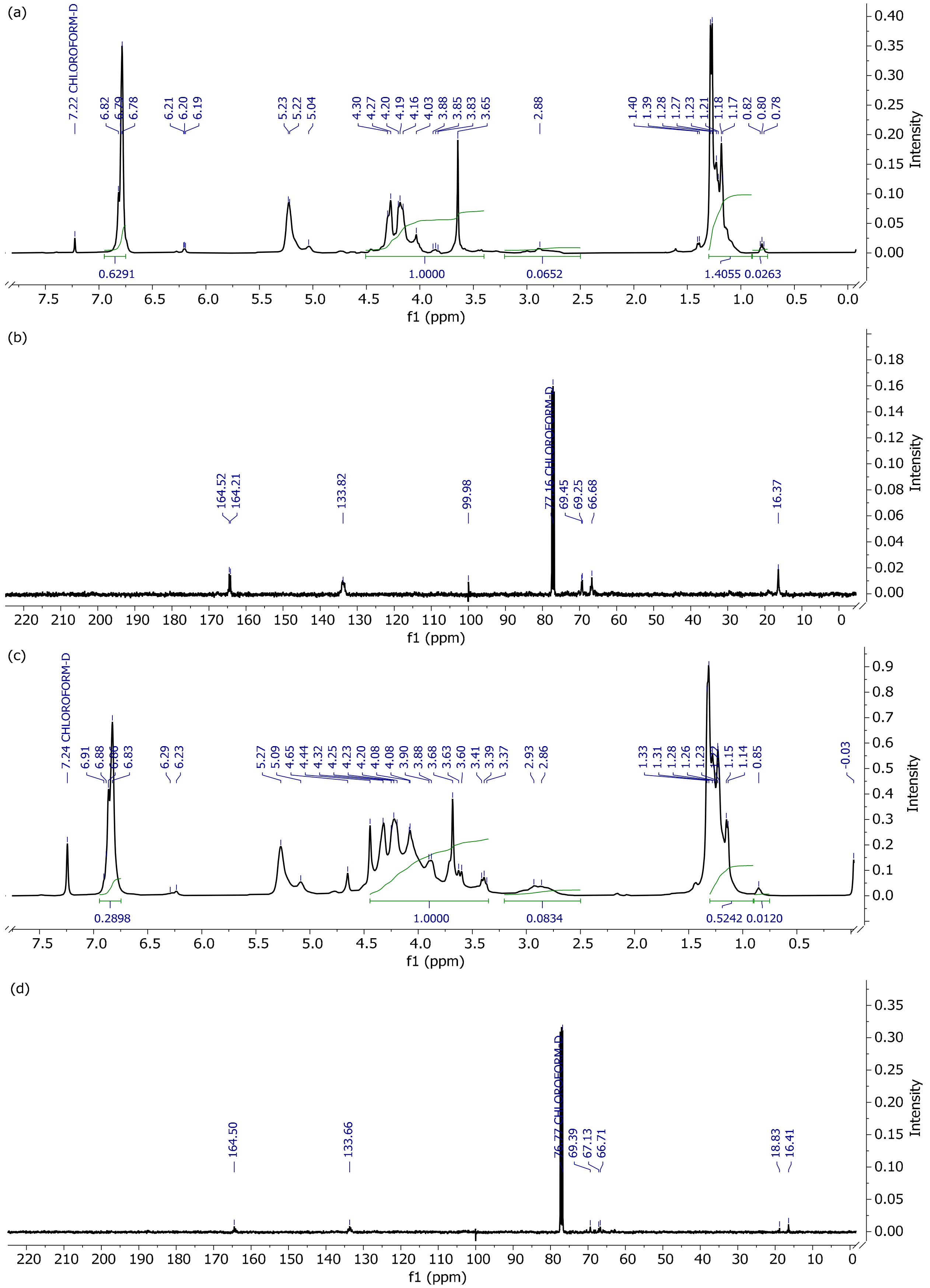
(a)
7.22 CHLOROFORM-D
6.82
6.79
6.78
6.21
6.20
6.19
5.23
5.22
5.04
4.30
4.27
4.20
4.19
4.16
4.03
3.88
3.85
3.83
3.65
2.88
1.40
1.39
1.28
1.27
1.23
1.21
1.18
1.17
0.82
0.80
0.78
0.6291
1.0000
0.0652
1.4055
0.0263
f1 (ppm)
Intensity
(b)
164.52
164.21
133.82
99.98
77.16 CHLOROFORM-D
69.45
69.25
66.68
16.37
f1 (ppm)
Intensity
(c)
7.24 CHLOROFORM-D
6.91
6.88
6.86
6.83
6.29
6.23
5.27
5.09
4.65
4.44
4.32
4.25
4.23
4.20
4.08
4.08
3.90
3.88
3.68
3.63
3.60
3.41
3.39
3.37
2.93
2.86
1.33
1.31
1.28
1.26
1.23
1.15
1.14
0.85
-0.03
0.2898
1.0000
0.0834
0.5242
0.0120
f1 (ppm)
Intensity
(d)
164.50
133.66
76.77 CHLOROFORM-D
69.39
67.13
66.71
18.83
16.41
f1 (ppm)
Intensity

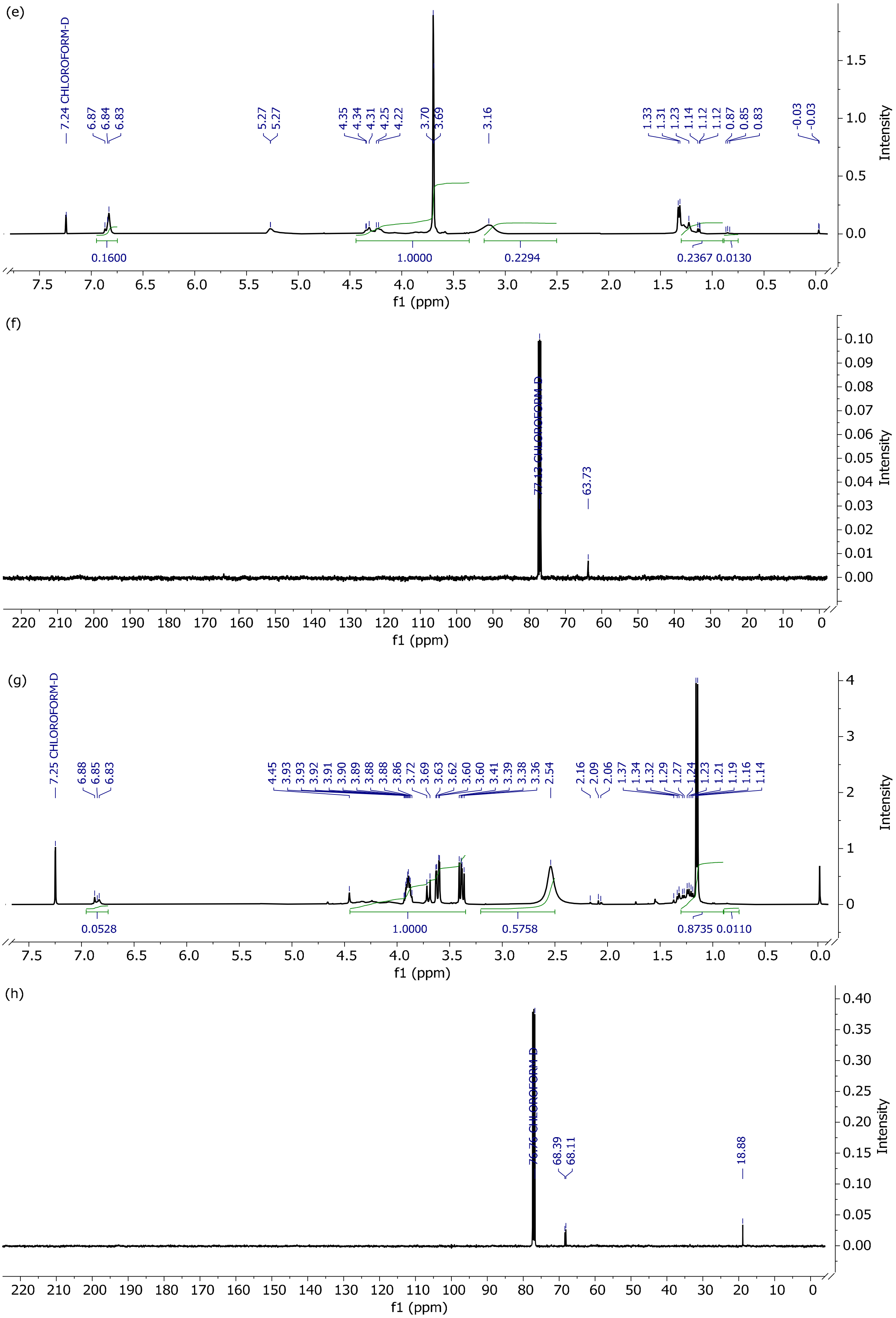

Figure S1. ^{1}H and ^{13}C NMR spectra of p-EGM:AA:[CPDT] copolymers synthesized in the presence of different concentrations of the RAFT agent CPDT: (a,b) 10 mM; (c,d) 30 mM; (e,f) 50 mM; and (g,h) 80

mM. Panels (a), (c), (e), and (g) show the ^{1}H NMR spectra, whereas panels (b), (d), (f), and (h) show the ^{13}C NMR spectra.
