# Supplementary material for: Structural Evolution of RAFT-Modified Unsaturated Polyester Copolymers: Effects of CPDT Concentration, Acidic Comonomer Structure, and Polyester Matrix Architecture
Source: Molecules. 2026 Aug 24;31(17):2958. doi: 10.3390/molecules31172958 (PMC13566969; doi:10.3390/molecules31172958)
Supplement: Supplementary file 1 [file molecules-31-02958-s001.zip › Figure S2.pdf]

(g)

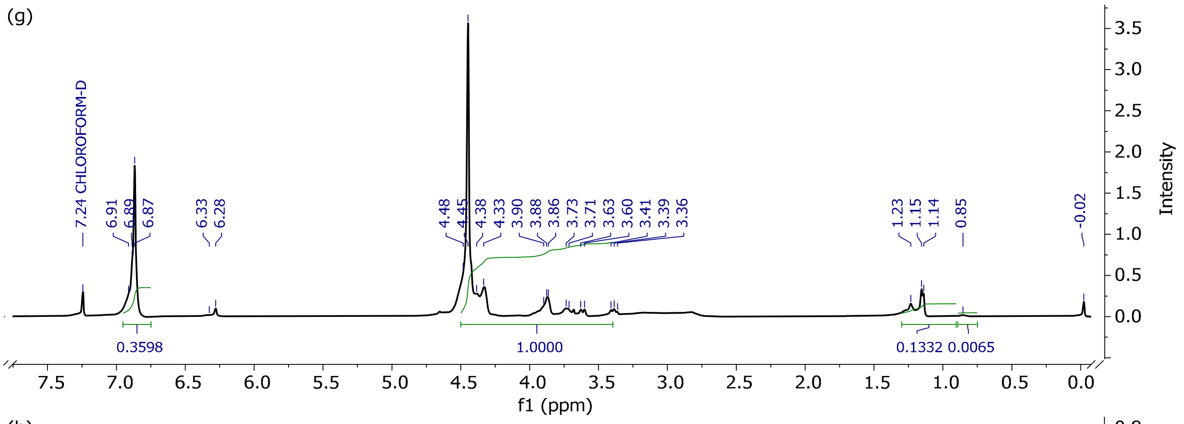
7.24 CHLOROFORM-D
6.91
6.89
6.87
6.33
6.28
4.48
4.45
4.38
4.33
3.90
3.88
3.86
3.73
3.71
3.63
3.60
3.41
3.39
3.36
1.23
1.15
1.14
0.85
-0.02
0.3598
1.0000
0.1332 0.0065
Intensity
f1 (ppm)

(h)

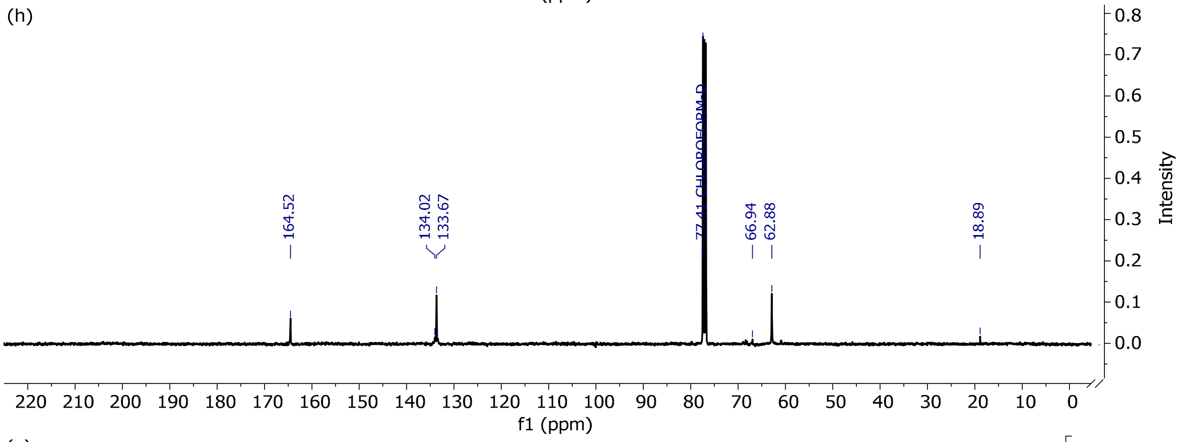
164.52
134.02
133.67
77.41 CHLOROFORM-D
66.94
62.88
18.89
Intensity
f1 (ppm)

(a)

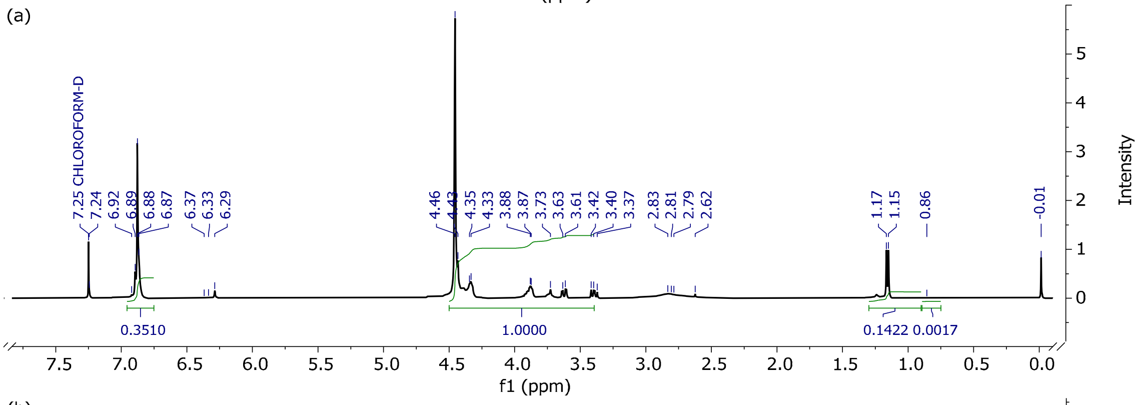
7.25 CHLOROFORM-D
7.24
6.92
6.89
6.88
6.87
6.37
6.33
6.29
4.46
4.43
4.35
4.33
3.88
3.87
3.73
3.63
3.61
3.42
3.40
3.37
2.83
2.81
2.79
2.62
1.17
1.15
0.86
-0.01
0.3510
1.0000
0.1422 0.0017
Intensity
f1 (ppm)

(b)

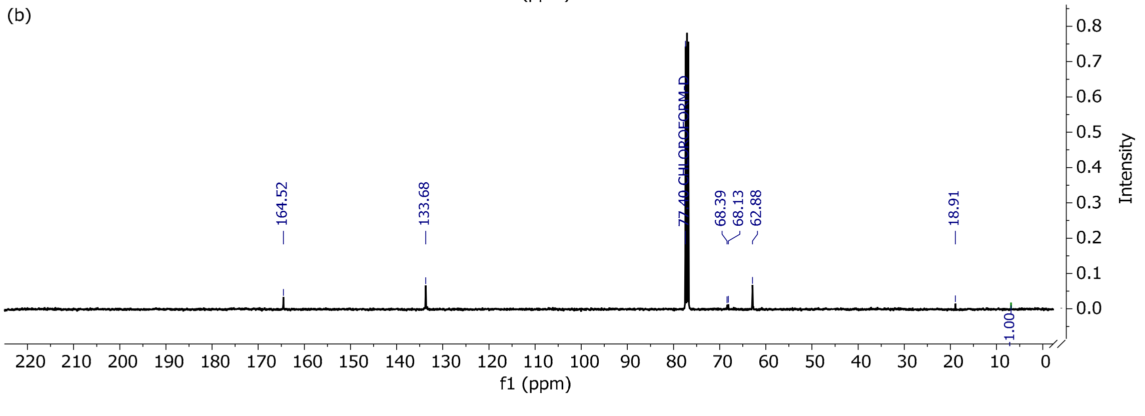
164.52
133.68
77.40 CHLOROFORM-D
68.39
68.13
62.88
18.91
1.00
Intensity
f1 (ppm)

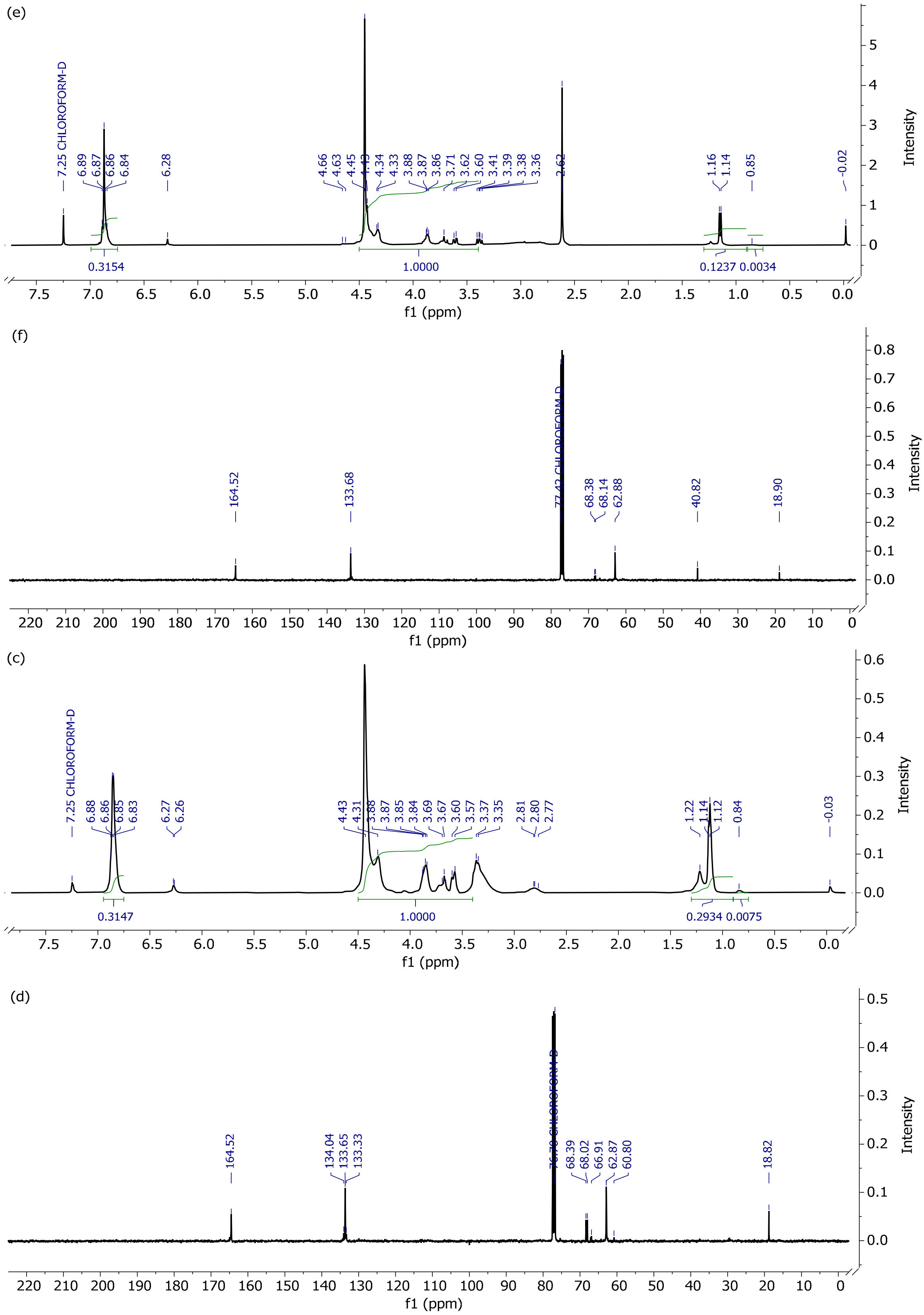


**Figure S2.** $^{1}H$ and $^{13}C$ NMR spectra of p-EGM:MAA:[CPDT] copolymers synthesized in the presence of different concentrations of the RAFT agent CPDT: (a,b) 10 mM; (c,d) 30 mM; (e,f) 50 mM; and (g,h) 80 mM. Odd-numbered panels correspond to $^{1}H$ NMR spectra, whereas even-numbered panels correspond to $^{13}C$ NMR spectra
