# Supplementary material for: Structural Evolution of RAFT-Modified Unsaturated Polyester Copolymers: Effects of CPDT Concentration, Acidic Comonomer Structure, and Polyester Matrix Architecture
Source: Molecules. 2026 Aug 24;31(17):2958. doi: 10.3390/molecules31172958 (PMC13566969; doi:10.3390/molecules31172958)
Supplement: Supplementary file 1 [file molecules-31-02958-s001.zip › Figure S3.pdf]

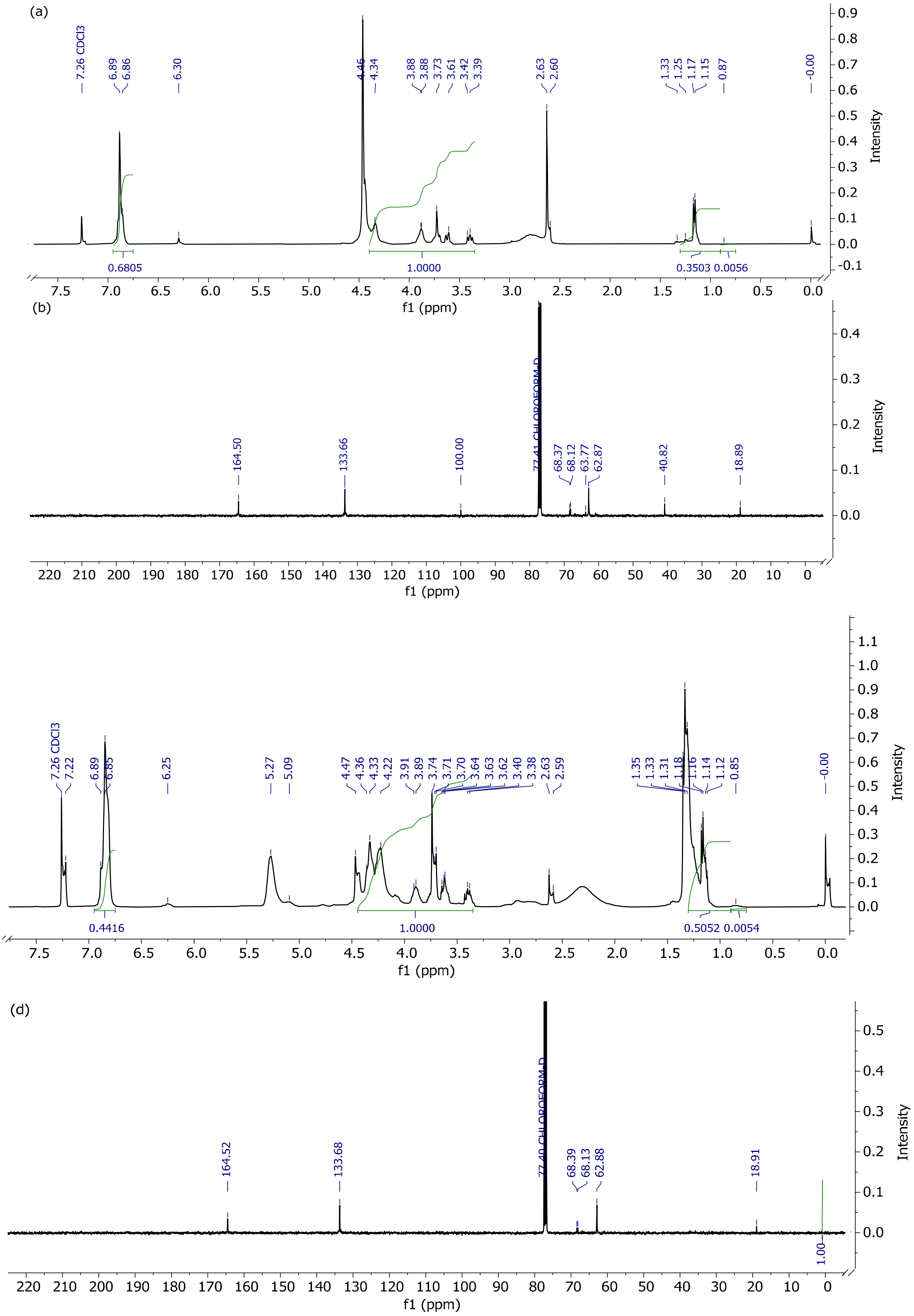

(a)
7.26 CDCl3
6.89
6.86
6.30
4.46
4.34
3.88
3.88
3.73
3.61
3.42
3.39
2.63
2.60
1.33
1.25
1.17
1.15
0.87
-0.00
0.6805
1.0000
0.3503
0.0056
f1 (ppm)
Intensity
(b)
164.50
133.66
100.00
77.41 CHLOROFORM-D
68.37
68.12
63.77
62.87
40.82
18.89
f1 (ppm)
Intensity
7.26 CDCl3
7.22
6.89
6.85
6.25
5.27
5.09
4.47
4.36
4.33
4.22
3.91
3.89
3.74
3.71
3.70
3.64
3.63
3.62
3.40
3.38
2.63
2.59
1.35
1.33
1.31
1.18
1.16
1.14
1.12
0.85
-0.00
0.4416
1.0000
0.5052
0.0054
f1 (ppm)
Intensity
(d)
164.52
133.68
77.40 CHLOROFORM-D
68.39
68.13
62.88
18.91
1.00
f1 (ppm)
Intensity

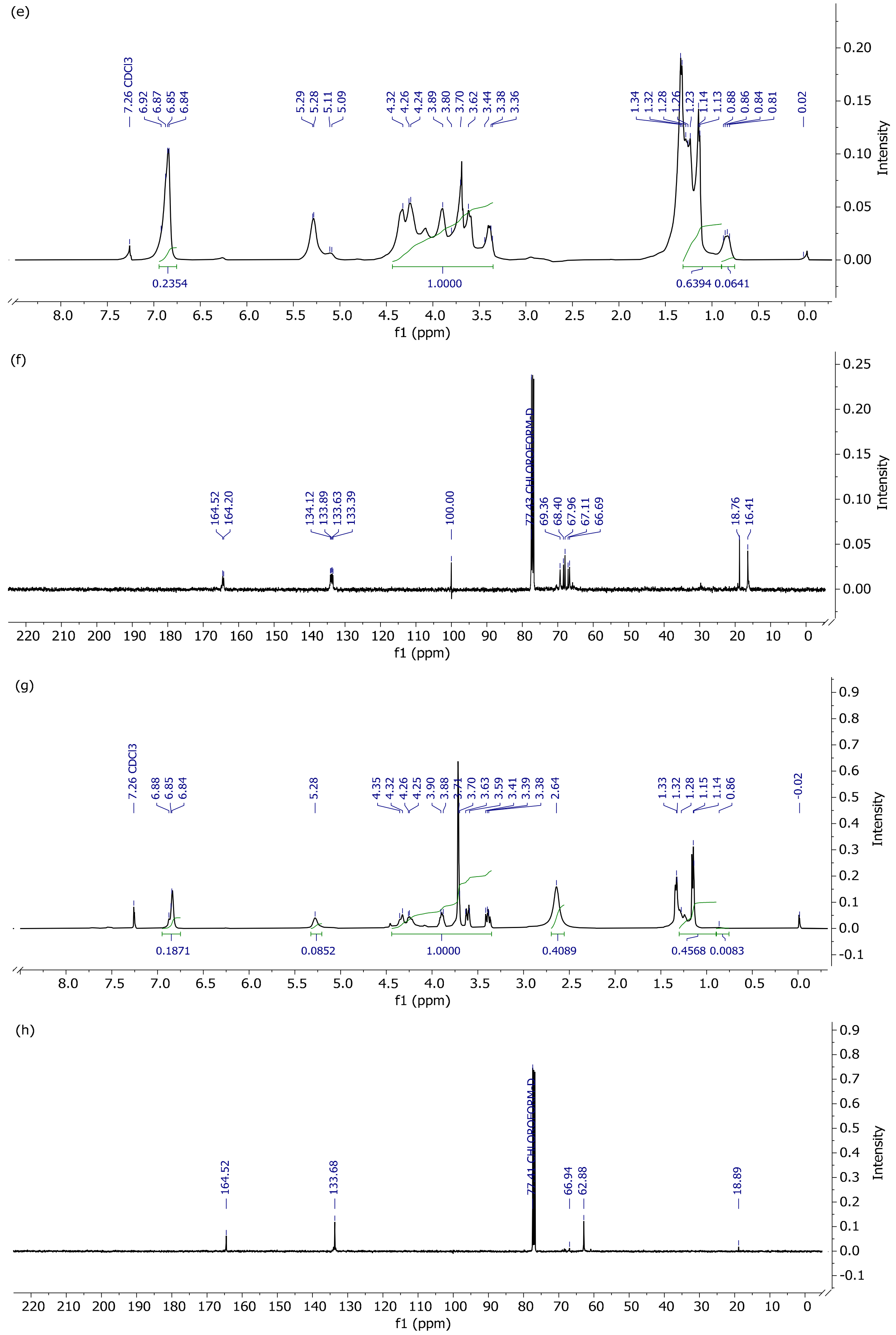

Figure S3. ^{1}H and ^{13}C NMR spectra of p-PGM:MAA:[CPDT] copolymers synthesized in the presence of different concentrations of the RAFT agent CPDT: (a,b) 10 mM; (c,d) 30 mM; (e,f) 50 mM; and (g,h) 80

mM. Odd-numbered panels correspond to ^{1}H NMR spectra, whereas even-numbered panels correspond to ^{13}C NMR spectra
