# Supplementary material for: Structural Evolution of RAFT-Modified Unsaturated Polyester Copolymers: Effects of CPDT Concentration, Acidic Comonomer Structure, and Polyester Matrix Architecture
Source: Molecules. 2026 Aug 24;31(17):2958. doi: 10.3390/molecules31172958 (PMC13566969; doi:10.3390/molecules31172958)
Supplement: Supplementary file 1 [file molecules-31-02958-s001.zip › Scheme S1.pdf]

a) Initiation

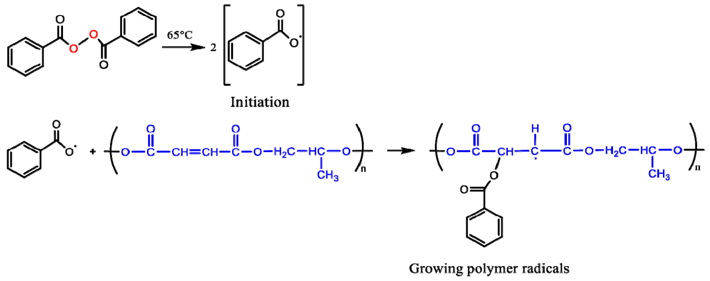

b) Propagation

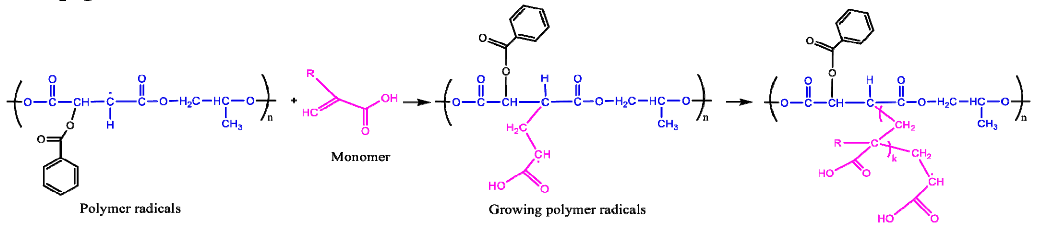

c) Addition to RAFT agent

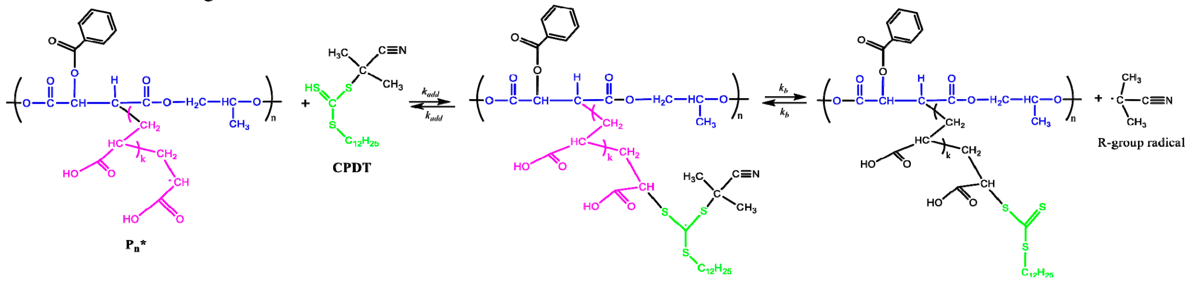

d) RAFT radical intermediate

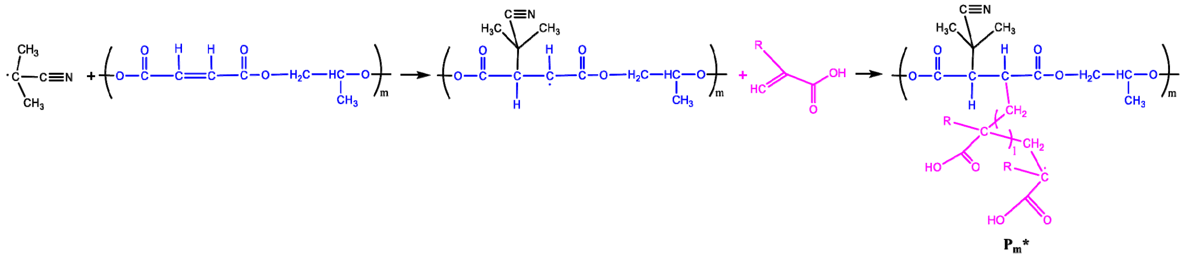

e) Dynamic equilibrium

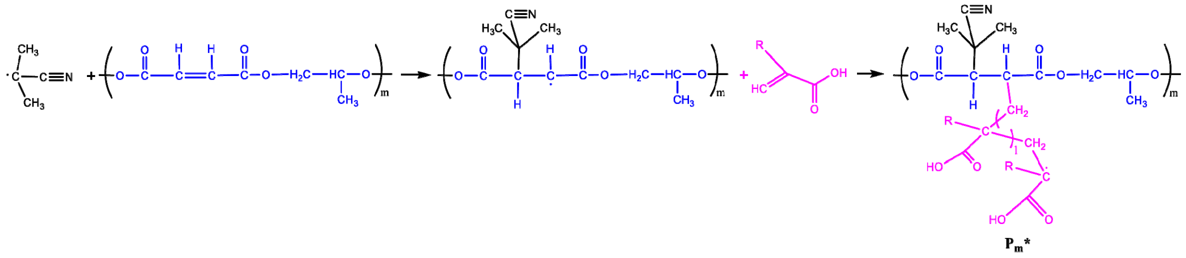

e) Dynamic equilibrium

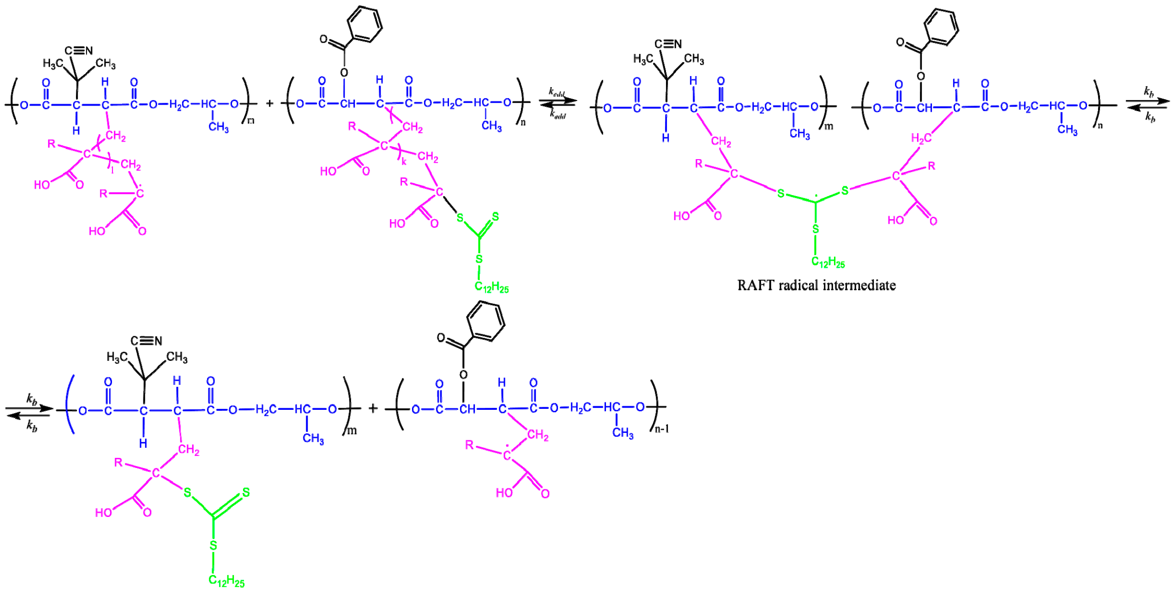

Scheme S1. Synthetic route for the preparation of the investigated RAFT-modified unsaturated polyester copolymers (p-EGM:AA:[CPDT], p-EGM:MAA:[CPDT], and p-PGM:MAA:[CPDT])
